# Determining the efficacy and safety of acupuncture for the treatment of menstrual migraine: an updated systematic review and meta-analysis

**DOI:** 10.3389/fneur.2025.1673321

**Published:** 2025-09-17

**Authors:** Qiqi Wu, Lijuan Fan, Danhui Wu, Hong Gao, Dexiong Han, Hantong Hu, Lala Qian

**Affiliations:** ^1^Department of Acupuncture, Moxibustion and Massage, Wenzhou Central Hospital, Wenzhou, China; ^2^Department of Comprehensive Rehabilitation, Zhejiang Rehabilitation Medical Center, Hangzhou, China; ^3^Department of Acupuncture, Moxibustion and Massage, Quzhou Hospital of Traditional Chinese Medicine, Quzhou, China; ^4^Department of Acupuncture and Moxibustion, The Third Affiliated Hospital of Zhejiang Chinese Medical University, Hangzhou, China

**Keywords:** menstrual migraine, acupuncture, systematic review, meta-analysis, headache

## Abstract

**Background:**

Menstrual migraine (MM) is a common type of headache linked to hormonal fluctuations during the menstrual cycle, remains challenging to treat due to the limited efficacy of current therapies. While acupuncture shows potential as a therapeutic option for MM, the existing evidence does not clearly support its routine clinical use. This protocol for a systematic review (SR) and meta-analysis seeks to gather and evaluate recent clinical evidence regarding the effectiveness and safety of acupuncture in treating MM.

**Methods:**

A comprehensive search was conducted across multiple databases from their inception to April 2025, including PubMed, Medline, Ovid, Embase, ScienceDirect, the Chinese National Knowledge Infrastructure (CNKI), Wanfang, the Chinese Biomedical Literature (CBM) database, and the VIP Database. This was complemented by regular updates from trial registries such as the Cochrane Central Register of Controlled Trials (CENTRAL) and the WHO International Clinical Trials Registry Platform (ICTRP), which target unpublished and ongoing randomized controlled trials (RCTs). Eligible studies were RCTs comparing acupuncture with Western medicine, herbal remedies, sham acupuncture, or no intervention for the management of MM. Primary outcomes included the pain intensity [Visual Analog Scale (VAS)], the frequency of migraine attacks (FM), and duration of migraine (DM). Secondary outcomes encompassed response rate, Headache Impact Test-6 (HIT-6), the Menstrual Headache Index (MHI), the serum levels of 5-hydroxytryptamine (5-HT), and adverse events. Analyses involved meta-analysis, subgroup comparisons, publication bias detection, sensitivity testing, risk-of-bias evaluations, and profiling of adverse events. The quality of evidence was judged according to the Grading of Recommendations Assessment, Development, and Evaluation (GRADE) criteria.

**Results:**

A total of 39 RCTs comprising 2,584 participants were included. Compared with control interventions, acupuncture significantly reduced VAS scores, decreased the frequency and duration of migraine attacks, and improved response rates, as well as HIT-6 and MHI scores. Additionally, acupuncture was associated with increased serum 5-HT levels. Meta-analytic findings indicated that acupuncture demonstrated a favorable safety profile in the treatment of MM.

**Conclusion:**

Findings suggest that acupuncture for MM produced the most notable reductions in migraine severity. Nevertheless, the GRADE assessment indicated low overall certainty of the evidence, with considerable heterogeneity present in multiple pooled analyses. Therefore, well-designed, large-scale RCTs are urgently required to strengthen the evidence base.

**Systematic review registration:**

https://www.crd.york.ac.uk/PROSPERO/view/CRD42022367446.

## Introduction

Migraine is a prevalent chronic neurological disorder characterized by recurrent attacks of headache and associated sensory disturbances. According to the latest Global Burden of Disease Study (GBD 2021), it remains one of the most common neurological conditions worldwide and a leading cause of disability, particularly among young and middle-aged women (1). The pathophysiology of migraine is now primarily understood as a disorder of brain function, involving complex mechanisms such as cortical spreading depression, trigeminovascular system activation, and central sensitization, rather than a primary vascular etiology (2). The International Headache Society characterizes migraine as a recurrent headache lasting between 4 and 72 h, typically presenting with at least two of the following: pulsating pain, one-sided location, worsening with physical activity, and moderate to severe intensity. Additionally, it must be accompanied by at least one of the following symptoms: nausea or vomiting, or heightened sensitivity to light and sound (3). Although its precise etiology remains complex, contemporary frameworks characterize migraine as a disorder of brain function, moving beyond the traditional neurovascular model. A recent synthesis by Raggi et al. (2) delineates key hallmarks of migraine, including cortical spreading depression, trigeminovascular system activation, and central sensitization, providing a more integrated pathophysiological model. Globally, it affects approximately 18.9% of women (4), with menstrual migraine (MM) representing a subtype that occurs in relation to the menstrual cycle.

MM episodes frequently begin shortly before menstruation, intensify during the menstrual phase, and subside afterward. The frequency and severity of attacks often diminish during pregnancy or following menopause, likely due to hormonal stabilization (5, 6). The relationship between ovarian hormones and migraine is complex and extends beyond the reproductive years. Recent evidence highlights that the perimenopausal period, characterized by erratic hormonal fluctuations, can significantly influence migraine course, often leading to an increase in frequency and severity before eventual attenuation post-menopause (7). MM-related headaches are typically more intense than those of other migraine subtypes, tend to recur, and can persist for 4 to 5 days—often aligning with the menstrual period. Common accompanying symptoms include nausea, sensitivity to light, mood disturbances such as irritability and depression, spontaneous perspiration, neck rigidity, and vomiting (8–10).

Research suggests a strong correlation between MM and the ovarian hormonal cycle (11). Approximately 60% of women with migraine report that their attacks are temporally related to menstruation (12). Although the exact pathophysiology of MM remains incompletely understood, numerous studies suggest that cyclical fluctuations in sex hormones—particularly estrogen—interact with the unique physiological and genetic characteristics of female migraine patients. These hormonal shifts are believed to influence both the structure and function of neural cells, potentially exerting either analgesic or pro-nociceptive effects depending on internal and external environmental factors (13, 14).

The diagnosis and clinical management of MM remain suboptimal, with a significant portion of patients not achieving adequate relief with conventional treatments (15). Present therapeutic approaches are largely adapted from general migraine treatment strategies, encompassing both acute interventions and preventive measures (16, 17). Acute therapies aim to halt ongoing migraine episodes, whereas preventive approaches seek to lower their frequency, intensity, and duration (16, 17). Common treatments include pharmacologic agents—such as non-steroidal anti-inflammatory drugs (NSAIDs), triptans, gepants, and ditans for acute care; and beta-blockers, anticonvulsants, antidepressants, and calcitonin gene-related peptide (CGRP) pathway targeting monoclonal antibodies and gepants for prevention (16–18), alongside non-drug interventions like neuromodulation devices, cognitive behavioral therapy, and biobehavioral training (16, 19, 20). Nevertheless, pharmacologic therapies in Western medicine often exhibits considerable interindividual variability and typically requires prolonged administration. Extended use can increase the risk of relapse and adverse effects, particularly gastrointestinal complications. Furthermore, excessive reliance on analgesics or migraine-specific drugs may exacerbate headache frequency, contributing to medication overuse headaches (21, 22). As a result, minimizing drug-induced side effects while enhancing patient quality of life and symptom control has become a central focus. In this context, complementary and alternative medicine (CAM) is being explored as a potential adjunct or alternative therapeutic option for individuals with MM.

Acupuncture has been consistently identified as a promising CAM intervention for managing MM. Its therapeutic applications in MM encompass both acute relief during migraine episodes and long-term prophylaxis aimed at decreasing the frequency, intensity, and duration of attacks. From a biomedical standpoint, the underlying mechanisms are believed to primarily involve modulation of the trigeminovascular system. Empirical evidence has demonstrated acupuncture’s regulatory influence on key trigeminal system-associated factors, including cortical spreading depression, astrocyte activity, and neurogenic kinins (23–26). Importantly, a growing body of clinical research support the effectiveness of acupuncture in managing MM.

Although a prior systematic review (SR) and meta-analysis (27) attempted to assess the therapeutic efficacy of acupuncture for MM but concluded that, acupuncture could not be recommended due to insufficient supporting evidence. Consequently, definitive conclusions regarding acupuncture’s clinical utility in MM remain lacking. It is important to highlight that this earlier review (27) included only randomized controlled trials (RCTs) published before May 1, 2019, resulting in a limited dataset of just 13 RCTs. In light of the substantial rise in RCTs published over the past 6 years, there is a pressing need to incorporate this newer evidence into an updated SR and meta-analysis. Therefore, the present study aims to synthesize current findings to rigorously assess the efficacy and safety of acupuncture for the prophylactic management of MM.

## Methods

This SR and meta-analysis adhered to the Preferred Reporting Items for Systematic Reviews and Meta-Analyses (PRISMA) 2020 statement (28), as detailed in Appendix S1. The study protocol was prospectively registered in the PROSPERO database under registration number CRD42022367446. To enhance the reporting quality of the acupuncture interventions, the Standards for Reporting Interventions in Clinical Trials of Acupuncture (STRICTA) checklist (29) was also consulted.

### Database and search strategy

Two reviewers (QQW and LLQ) independently conducted comprehensive searches across multiple databases from their inception through April 2025. The databases included Embase, Ovid, Medline, PubMed, ScienceDirect, the Chinese National Knowledge Infrastructure (CNKI), Wanfang Data, the Chinese Biomedical Literature Database (CBM), and the VIP Database for Chinese Technical Periodicals (VIP). To ensure completeness, the search was supplemented by periodic manual checks for unpublished and ongoing RCTs within the Cochrane Central Register of Controlled Trials (CENTRAL) and the WHO International Clinical Trials Registry Platform (ICTRP). Only original research articles published in English or Chinese were considered eligible for inclusion.

A thorough literature search was conducted in PubMed using a structured combination of search terms. The first category included migraine-related terms: “migraine disorders,” “migraine”, “menstruation,” “menstruation-related migraine,” “menses,” “menstrually,” “menstrual,” and “menstrual migraine,” searched within titles or abstracts. The second group comprised acupuncture-related terms: “acupuncture,” “acupuncture therapy,” “scalp acupuncture,” “auricular acupuncture,” “ear acupuncture,” “warm needle,” “warming-needle moxibustion,” “fire acupuncture,” “acupoint catgut embedding,” “electroacupuncture,” “electro-acupuncture,” “manual acupuncture,” “intradermal needle,” “triangular needle,” “pricking blood,” “bloodletting,” “plum blossom needling,” and “acupoint injection,” also limited to titles or abstracts. The third set targeted study design identifiers, including: “clinical trial,” “randomized clinical trial,” “controlled clinical trial,” and “randomized controlled trial”, within the same fields. The final query combined all three categories using the Boolean operator “#1 AND #2 AND #3.” Detailed search strategies for each database are presented in Appendix S2.

### Inclusion criteria

#### Study design

Only RCTs available in the public domain were included. Eligible studies must clearly describe the method of randomization or explicitly mention the use of random allocation within the published text.

#### Participants

1. Individuals diagnosed with MM in accordance with established clinical guidelines;
2. Exclusion of subjects with comorbid severe illnesses;
3. Exclusion of participants previously diagnosed with other headache disorders, including tension-type headaches, chronic migraine, cluster headaches, other primary headaches, or secondary headaches arising from ear, nose, and throat (ENT) conditions or intracranial abnormalities; and
4. No restrictions were applied based on age, sex, geographic location, race, disease duration, or symptom severity.

#### Interventions

Studies investigating acupuncture-related interventions were considered, including acupoint injection, moxibustion, electroacupuncture, auricular acupuncture, abdominal acupuncture, and body acupuncture. Interventions could be administered alone or alongside the same active treatments provided to the control group.

#### Control conditions

1. Accepted therapeutic options for MM, such as conventional Western medications (oral, intravenous infusion, or injection) or traditional Chinese medicine, were included;
2. Studies using waiting list controls or no treatment groups were eligible; and
3. Sham acupuncture was also considered an acceptable comparator.

### Exclusion criteria

Studies were excluded under the following conditions: if the control group received any form of acupuncture. Duplicate publications, animal studies, conference abstracts, and trials lacking sufficient data for analysis were also omitted. Furthermore, trials that merely referenced the term “random” without providing a clear description of the randomization procedure were excluded from the analysis.

### Outcome measures

Primary outcomes consisted of the pain intensity assessed by Visual Analoge Scale (VAS), the frequency of migraine attacks (FM), and duration of migraine (DM).

Secondary outcomes included response rate, Headache Impact Test-6 (HIT-6) scores, the menstrual headache index (MHI), serum concentrations of 5-hydroxytryptamine (5-HT), and incidence of adverse events. Reported adverse events encompassed acupuncture-related complications such as syncope, needle deformation or retention, needle breakage, localized infection, and hematoma formation.

### Study selection

Two reviewers (QQW and LLQ) independently screened the titles and abstracts of retrieved publications to identify eligible RCTs. In cases of overlapping or duplicate data, only the most recent or most comprehensive study was retained. Full texts of the preliminarily selected records were then reviewed to capture additional relevant studies potentially missed in the initial search. The same two reviewers subsequently assessed the full texts against the predefined inclusion criteria. Discrepancies were resolved through discussion, and when needed, a third reviewer (HTH) was consulted to achieve consensus.

### Data extraction and management

Two authors (QQW and LJF) independently extracted data, including study characteristics, participant demographics, intervention specifics, and outcome variables. Disagreements in data extraction were resolved through discussion, with input from a third author (HTH) if required.

### Methodological evaluation and quality assessment

The methodological quality of the included studies was evaluated using the Cochrane risk-of-bias tool 2.0 (RoB 2.0), following the criteria specified in The Cochrane Handbook for Systematic Reviews of Interventions (30). For each outcome, the certainty of evidence and potential risk of bias were assessed using the Grading of Recommendations Assessment, Development, and Evaluation (GRADE) framework.

Two authors (QQW and LLQ) independently evaluated the methodological quality of the included studies. Any disagreements regarding the appraisal of evidence quality were resolved through discussion, with the final consensus reached in consultation with a third reviewer (HTH).

### Data analysis

Statistical analyses were performed using Review Manager (RevMan) software, version 5.4. For binary outcomes such as response rate and incidence of adverse events, odds ratios (ORs) with 95% confidence intervals (CIs) were calculated. Continuous outcomes—including VAS, FM, and DM (analyzed using standardized mean differences, SMDs) as well as HIT-6, MHI, and 5-HT (analyzed using mean differences, MDs)—were reported with corresponding 95% CIs (31, 32). A fixed-effect model was applied when heterogeneity was minimal (*p* > 0.1 and I^2^ < 50%); otherwise, a random-effects model was used to pool the data (33).

To evaluate the robustness of the findings, sensitivity analyses were conducted to evaluate the robustness of the pooled results for the primary outcomes (VAS, FM, and DM) using the leave-one-out method. Funnel plots and Egger’s test were used to detect publication bias, and a *p*-value < 0.05 was considered statistically significant (34).

## Results

### Trial identification

**Figure 1:**
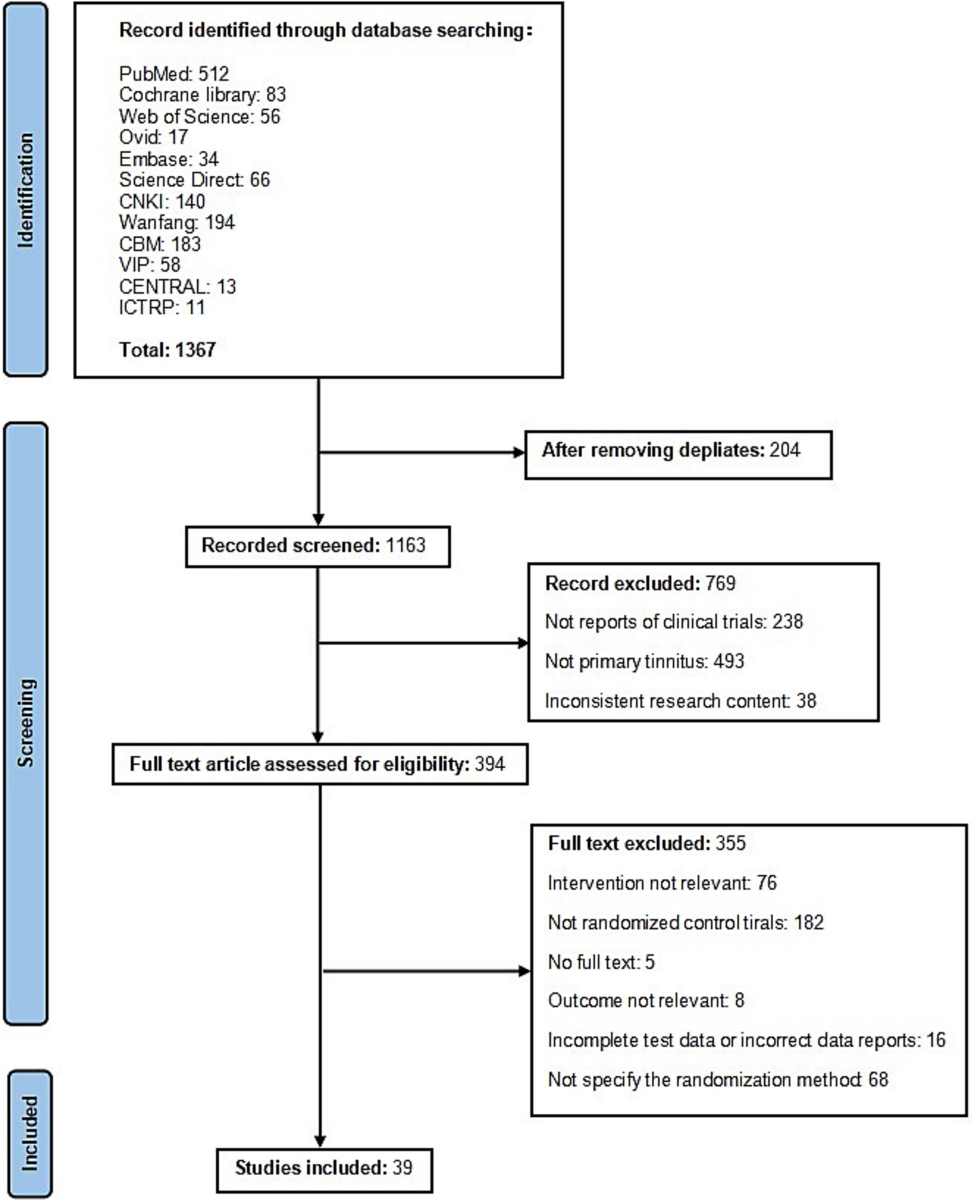
PRISMA flowchart of the process used to retrieve relevant articles from the literature.

The literature search yielded 1,367 potentially relevant records. After removing 204 duplicates, titles and abstracts of the remaining articles were screened, resulting in 394 studies considered for further evaluation. Following full-text assessment, 355 RCTs were excluded due to non-compliance with the inclusion criteria. Ultimately, 39 studies were deemed eligible and incorporated into the final analysis (Figure 1).

### Characteristics of the included studies

Table 1 presents the key characteristics of the 39 RCTs included in this analysis (total participants: *n* = 2,584; intervention group: *n* = 1,313; control group: *n* = 1,271). Among these, 16 studies (35–51) involved filiform needle, one trial (52) used balancing acupuncture, one study (53) involved body acupuncture with auricular acupressure, one trial (54) involved electroacupuncture and triple puncture, one trial (55) used scalp penetration acupuncture with body acupuncture, one study (56) involved pricking bloodletting, one trial (57) involved shuitu acupoint transcutaneous electric nerve stimulation with acupoint injection, one trial (58) used acupoint injection, one study (59) involved acupuncture with mild moxibustion, two trials (46, 60) used filiform needle with traditional Chinese medicine, two trials (61, 62) involved warm acupuncture treatment, one study (63) used thumb-tack auricular acupuncture with traditional Chinese medicine, two trials (64, 65) involved scalp acupuncture stimulation with moxibustion of Zhuang medicated thread, one trial (66) involved thermal moxibustion, one trial (67) involved auricular point pressing-bean, one trial (68) involved bloodletting at auricular dorsal vein with auricular point sticking, two studies (69, 70) involved auricular comprehensive therapy, one trial (71) used Jiejie acupuncture, one trial (72) involved eye acupuncture, and one trial (73) involved electroacupuncture.

**Table 1:** The characteristics of the randomized controlled trials (RCTs) included in this meta-analysis.

Included trials	Eligibility criteria	Countries	Intervention and treatment		Sample and characteristics (male female, age, disease duration)		Outcomes
			Trial	Control	Trial	Control	
M Linde (2005) (35)	CDCHDCNFP	Sweden	Types of acupuncture: filiform needle Deqi achieved?: UN Acupuncturist background?: YES Needle retention time: 30 min Frequency of sessions and treatment courses: D-8, D-5, D-3 (±1d) pre-menses per cycle × 3 Number of treatment sessions: 9	Sham acupuncture	15; AGE: (mean: 35.2 ± 7.5y); Disease duration: -	13; AGE: (mean: 37.4 ± 8.6y); Disease duration: -	FM VAS
Zhouhong Sun (2008) (36)	DECCME	China	Types of acupuncture: filiform needle Deqi achieved?: UN Acupuncturist background?: UN Needle retention time: 30 min Frequency of sessions and treatment courses: qod × 5/cycle × 3 Number of treatment sessions: 15	Western medicine (ibuprofen capsule)	42; AGE: 17−45y; Disease duration: 3 m−15y	42; AGE: 17−45y; Disease duration: 3 m−15y	Response rate
Fangrong Liu (2009) (37)	DECCME	China	Types of acupuncture: filiform needle Deqi achieved?: YES Acupuncturist background?: UN Needle retention time: UN Frequency of sessions and treatment courses: qd × 7d/course × 4 (start: 1wk pre-menses) Number of treatment sessions: 28	Western medicine (aspirin)	30; AGE: 14−49y (mean: 26.4 ± 1.71y); Disease duration: (mean: 26.04 ± 0.51y)	30; AGE: 15−49y (mean: 25.3 ± 2.01y); Disease duration: (mean: 5.22 ± 1.01y)	VAS DM MHI Response rate
Guoxian Wu (2009) (38)	DECCME	China	Types of acupuncture: filiform needle Deqi achieved?: UN Acupuncturist background?: UN Needle retention time: UN Frequency of sessions and treatment courses: 1wk pre-menses → menses end (2 cycles/course × 2) Number of treatment sessions: UN	Herbal medicine	20; AGE: 15−49y (mean: 25.3 ± 2.01y); Disease duration: (mean: 5.22 ± 1.01 m)	20; AGE: 14−49y (mean: 26.4 ± 1.71y); Disease duration: (mean: 6.04 ± 0.51 m)	VAS DM MHI Response rate
Lina Chen (2011) (52)	ICHD-2 CBDEEH	China	Types of acupuncture: balancing acupuncture Deqi achieved?: YES Acupuncturist background?: UN Needle retention time: 0 min Frequency of sessions and treatment courses: 1/day for 14 days Number of treatment sessions: 14	Western medicine (celebrex)	43; AGE: 20−39y (mean: 29.81 ± 4.32y); Disease duration: 5d−8y (mean: 4.5 ± 2.5y)	26; AGE: 19−45y (mean: 28.43 ± 4.63y); Disease duration: 2d−9y (mean: 4.4 ± 2.7y)	Response rate
Hongli Ma (2012) (53)	DECCME	China	Types of acupuncture: body acupuncture + auricular acupressure Deqi achieved?: UN Acupuncturist background?: UN Needle retention time: 30 min-60 min Frequency of sessions and treatment courses: 3-course protocol: onset → qd × 3d; (2–3) D-3 pre-menses → qd × 6d Number of treatment sessions: 15	Western medicine (flunarizine hydrochloride capsules)	43; AGE: 18−39y; Disease duration: 1−4yb	42; AGE: 19−40y; Disease duration: 1−6y	Response rate VAS Adverse reactions
Ling Cao (2013) (54)	ICHD-2 CBDEEH	China	Types of acupuncture: electroacupuncture and triple puncture Deqi achieved?: YES Acupuncturist background?: UN Needle retention time: 30 min Frequency of sessions and treatment courses: start D + 26 post-menses: 1/day for 14 days Number of treatment sessions: 14	Western medicine (celebrex)	38; AGE: 18−43y (mean: 33.82 ± 4.34y); Disease duration: 3 m−11y	37; AGE: 21−42y (mean: 32.86 ± 4.85y); Disease duration: 2 m−13y	Response rate
Wenjing Lv (2014) (55)	DECCME	China	Types of acupuncture: scalp penetration acupuncture + body acupuncture Deqi achieved?: YES Acupuncturist background?: UN Needle retention time: 45 min Frequency of sessions and treatment courses: 3-course protocol: onset → qd × 7d; (2–3) D-7 pre-menses → qd × 14d Number of treatment sessions: 35	Western medicine (flunarizine hydrochloride capsules)	29; AGE: 19−42y (mean: 29.72 ± 5.33y); Disease duration: 2−9y (mean: 6.17 ± 1.81y)	28; AGE: 17−38y (mean: 28.57 ± 5.43y); Disease duration: 1−11y (mean: 6.42 ± 2.51y)	Response rate MHI Adverse reactions
Huilian Zhang (2014) (56)	DTRCMCTCM	China	Types of acupuncture: pricking bloodletting Deqi achieved?: UN Acupuncturist background?: UN Needle retention time: 0 min Frequency of sessions and treatment courses: D-7 pre-menses: 1/cycle, 3 cycles/course × 2 Number of treatment sessions: 6	Herbal medicine	49; AGE: 20−45y (mean 35 ± 6y); Disease duration: (mean: 6.04 ± 3.21y)	49; AGE: 22−44y (mean 35 ± 6y); Disease duration: (mean: 4.82 ± 2.34y)	VAS DM Response rate
Lihong Wang (1) (2015) (57)	ICHD-2	China	Types of acupuncture: shuitu acupoint transcutaneous electric nerve stimulation + acupoint injection Deqi achieved?: UN Acupuncturist background?: UN Needle retention time: 20 min Frequency of sessions and treatment courses: qod × 4 sessions/cycle, 3 cycles total Number of treatment sessions: 12	Western medicine (flunarizine hydrochloride capsules)	34; AGE: 18−38y (mean: 31.6 ± 9.6y); Disease duration: 6-33 m (mean: 11.7 ± 3.9 m)	34; AGE: 17−39y (mean: 32.6 ± 8.9y); Disease duration: 7-35 m (mean: 12.8 ± 7.9 m)	VAS FM DM Adverse reactions
Lihong Wang (2) (2015) (58)	ICHD-2	China	Types of acupuncture: acupoint injection Deqi achieved?: UN Acupuncturist background?: UN Needle retention time: 0 min Frequency of sessions and treatment courses: qod × 4 sessions/cycle, 3 cycles total Number of treatment sessions: 12	Western medicine (flunarizine hydrochloride capsules)	32; AGE: 17−40y (mean: 31 ± 10y); Disease duration: 6-34 m (mean: 11.8 ± 3.8 m)	32; AGE: 18−39y (mean: 33 ± 9y); Disease duration: 7-35 m (mean: 12.7 ± 7.9y)	VAS FM DM
SiJia Li (2016) (59)	DECCME Neurology	China	Types of acupuncture: acupuncture + mild moxibustion Deqi achieved?: YES Acupuncturist background?: UN Needle retention time: 30 min Frequency of sessions and treatment courses: 3-course protocol: (1) onset → qd × 6d; (2–3) D-10 pre-menses → qd × 14d (6d/wk) Number of treatment sessions: 18	Western medicine (ibuprofen sustained-release capsule)	30; AGE: 19−43y (mean: 29.77 ± 5.43y); Disease duration: 5-27 m (mean: 13.6 ± 5.26 m)	29; AGE: 20−41y (mean: 31.31 ± 5.65y); Disease duration: 5-36 m (mean: 12.48 ± 6.97 m)	VAS FM DM Response rate
Zhixia Liu (2016) (39)	OGITCWM	China	Types of acupuncture: filiform needle Deqi achieved?: YES Acupuncturist background?: UN Needle retention time: 0 min Frequency of sessions and treatment courses: D-3 pre-menses → qd → menses end (3 cycles) Number of treatment sessions: UN	Western medicine (flunarizine hydrochloride capsules)	30; AGE: 17−45y (mean: 30.23y); Disease duration: 1.5-9y (mean: 6.23y)	30; AGE: 18−45y (mean: 29.23y); Disease duration: 2–8.5y (mean: 6.11y)	Response rate
Xing Wang (2017) (40)	ICHD-3	China	Types of acupuncture: filiform needle Deqi achieved?: UN Acupuncturist background?: UN Needle retention time: UN Frequency of sessions and treatment courses: cycle × 3: Pre: qod × 3 (1wk pre); Menses: 2×/wk.; Other: BIW Number of treatment sessions: 27 (±6)	Sham acupuncture	17; AGE: (mean: 32.29 ± 7.02y); Disease duration: -	14; AGE: 17−45y (mean: 33.86 ± 7.86y); Disease duration: -	FM VAS HIT-6
Mengyuan Zhou (2017) (41)	ICHD-3	China	Types of acupuncture: filiform needle Deqi achieved?: YES Acupuncturist background?: UN Needle retention time: 30 min Frequency of sessions and treatment courses: cycle × 3: Pre: qod × 3 (1wk pre); Menses: 2×/wk.; Other: BIW Number of treatment sessions: 27 (±6)	Sham acupuncture	29; AGE: (mean: 33.1 ± 6.143y); Disease duration: (mean: 7.403 ± 6.3092y)	26; AGE: (mean: 33.27 ± 6.873y); Disease duration: (mean: 6.475 ± 4.6571y)	VAS FM DM Adverse reactions
Ziyi Yang (2018) (42)	ICHD-3	China	Types of acupuncture: filiform needle Deqi achieved?: YES Acupuncturist background?: UN Needle retention time: 30 min Frequency of sessions and treatment courses: cycle × 3: Pre: qod × 3 (1wk pre); Menses: 2×/wk.; Other: BIW Number of treatment sessions: 27 (±6)	Sham acupuncture	29; AGE: (mean: 33.1 ± 6.143y); Disease duration: (mean: 7.403 ± 6.3092y)	26; AGE: (mean: 33.27 ± 6.873y); Disease duration: (mean: 6.475 ± 4.6571y)	FM VAS HIT-6 Adverse reactions
Xianmin Yu (2018) (43)	ICHD-3	Canada	Types of acupuncture: filiform needle Deqi achieved?: YES Acupuncturist background?: UN Needle retention time: 20 min Frequency of sessions and treatment courses: 3 sessions/cycle × 3 cycles Number of treatment sessions: 9	Sham acupuncture	7; AGE: 22−52y; Disease duration: -	5; AGE: 22−52y; Disease duration: -	VAS DM
Xuefen Li (2018) (44)	DECCME GTCM	China	Types of acupuncture: filiform needle Deqi achieved?: YES Acupuncturist background?: UN Needle retention time: 30 min Frequency of sessions and treatment courses: 3-course protocol: (1) onset → qd × 7d; (2–3) D-7 pre-menses → qd × 14d Number of treatment sessions: 35	Western medicine (flunarizine hydrochloride capsules)	29; AGE: 20-39y (mean: 31.27 ± 5.34y); Disease duration: 0.42–2.25y (mean: 1.23 ± 0.44y)	29; AGE: 19-38y (mean: 29.75 ± 6.12y); Disease duration: 0.5–2.5y (mean: 1.12 ± 0.58y)	VAS MHI 5-HT Response rate Adverse reactions
Xuefen Li (2019) (45)	DECCME GTCM	China	Types of acupuncture: filiform needle Deqi achieved?: YES Acupuncturist background?: UN Needle retention time: 30 min Frequency of sessions and treatment courses: qd × 10d/course × 3 courses Number of treatment sessions: 30	Western medicine (flunarizine hydrochloride capsules)	30; AGE: (mean: 35.5 ± 4.1y); Disease duration: (mean: 12.3 ± 1.4 m)	30; AGE: (mean: 36.2 ± 2.9y); Disease duration: (mean: 11.9 ± 2.6 m)	5-HT Response rate
Zhihui Liu (2019) (46)	Unclear	China	Types of acupuncture: filiform needle + herbal medicine Deqi achieved?: UN Acupuncturist background?: UN Needle retention time: 30 min Frequency of sessions and treatment courses: qd × 5d/course × 3 courses Number of treatment sessions: 15	Herbal medicine	30; AGE: 31−46y (mean: 39.65 ± 4.55y); Disease duration: 2−11y (mean: 5.02 ± 1.73y)	30; AGE: 30−45y (mean: 38.25 ± 4.59y); Disease duration: 1−10y (mean: 4.26 ± 1.52y)	Response rate FM DM VAS Adverse reactions
Yutong Zhang (2021) (47)	ICHD-3	China	Types of acupuncture: filiform needle Deqi achieved?: YES Acupuncturist background?: UN Needle retention time: 30 min Frequency of sessions and treatment courses: cycle × 3: Pre: qod × 3 (1wk pre); Menses: 2×/wk.; Other: BIW Number of treatment sessions: 27 (±6)	Sham acupuncture	24; AGE: 18−50y (mean: 33.04 ± 6.43y); Disease duration: -	20; AGE: 18−50y (mean: 35.3 ± 9.43y); Disease duration: -	FM VAS
Yutong Zhang (2020) (48)	ICHD-3	China	Types of acupuncture: filiform needle Deqi achieved?: UN Acupuncturist background?: UN Needle retention time: UN Frequency of sessions and treatment courses: cycle × 3: Pre: qod × 3 (1wk pre); Menses: 2×/wk.; Other: BIW Number of treatment sessions: 27 (±6)	Sham acupuncture	17; AGE: 18−50y; Disease duration: -	14; AGE: 18−50y; Disease duration: -	FM VAS Adverse reactions
Jiao Bu (2020) (60)	CGDTM ICHD-3 COG	China	Types of acupuncture: filiform needle + herbal medicine Deqi achieved?: YES Acupuncturist background?: UN Needle retention time: 30 min Frequency of sessions and treatment courses: D-7 pre-menses → qd × 10d (1 course/cycle) × 3 cycles Number of treatment sessions: 30	Herbal medicine	35; AGE: 18−46y (mean: 29.83 ± 8.06y); Disease duration: (mean: 6.34 ± 1.64y)	34; AGE: 18−43y (mean: 28.68 ± 6.79y); Disease duration: (mean: 6.46 ± 1.75y)	VAS Response rate Adverse reactions
Lingyan Liu (2020) (61)	ICHD-3 DECCME GTCM	China	Types of acupuncture: warm acupuncture Deqi achieved?: YES Acupuncturist background?: UN Needle retention time: 30 min Frequency of sessions and treatment courses: D-7 pre-menses → qd × 7d/cycle × 3 cycles (adapt: stop at menses if no HA, continue if HA) Number of treatment sessions: >21	Western medicine (flunarizine hydrochloride capsules)	30; AGE: (mean: 32.43 ± 6.6y); Disease duration: (mean: 13.77 ± 6.12y)	30; AGE: (mean: 31.73 ± 6.66y); Disease duration: (mean: 12.87 ± 6.06y)	VAS FM DM HIT-6 Response rate Adverse reactions
Xuetong Tang (2020) (63)	OG GPCRNCM DECCME	China	Types of acupuncture: thumb-tack auricular acupuncture + herbal medicine Deqi achieved?: NO Acupuncturist background?: UN Needle retention time: 72 h Frequency of sessions and treatment courses: D-6 pre-menses: embed needles × 3d/ear, alternate ears q3d × 12d (1 course) × 3 cycles Number of treatment sessions: 12	Herbal medicine	31; AGE: 23−43y (mean: 32.45 ± 5.92y); Disease duration: -	30; AGE: 21−44y (mean: 31.7 ± 6.48y); Disease duration: -	MHI Response rate Adverse reactions
Jiaxing Yan (1) (2021) (64)	ICHD-3 DECCME	China	Types of acupuncture: scalp acupuncture stimulation + moxibustion of Zhuang medicated thread Deqi achieved?: YES Acupuncturist background?: UN Needle retention time: 30-50 min Frequency of sessions and treatment courses: D-7 pre-menses → qd × 10d/cycle × 3 cycles Number of treatment sessions: 30	Western medicine (flunarizine hydrochloride capsules)	35; AGE: 20−37y (mean: 29.1 ± 3.9y); Disease duration: 1−7y (mean: 3.5 ± 1.6y)	35; AGE: 19−36y (mean: 28.9 ± 4.3y); Disease duration: 1−7y (mean: 3.4 ± 1.5y)	Response rate VAS HIT-6
Jiaxing Yan (2) (2021) (65)	ICHD-3 DECCME	China	Types of acupuncture: scalp acupuncture stimulation + moxibustion of Zhuang medicated thread Deqi achieved?: YES Acupuncturist background?: UN Needle retention time: 30-50 min Frequency of sessions and treatment courses: D-7 pre-menses → qd × 10d/cycle × 3 cycles Number of treatment sessions: 30	Western medicine (flunarizine hydrochloride capsules)	30; AGE: 20−37y (mean: 28.9 ± 4.1y); Disease duration: 1−7y (mean: 3.7 ± 1.69y)	30; AGE: 19−36y (mean: 28.4 ± 4.67y); Disease duration: 1−7y (mean: 3.57 ± 1.57y)	VAS Response rate Adverse reactions
Liping Kuang (2022) (66)	ICHD-3	China	Types of acupuncture: thermal moxibustion Deqi achieved?: YES Acupuncturist background?: UN Needle retention time: UN Frequency of sessions and treatment courses: D-7 pre-menses → qd × 7d/cycle × 3 cycles Number of treatment sessions: 21	Western medicine (flunarizine hydrochloride capsules)	29; AGE: (mean: 31.93 ± 4.98y); Disease duration: (mean: 12.1 ± 3.3y)	30; AGE: (mean: 32.7 ± 5.45y); Disease duration: (mean: 11.93 ± 3.97y)	VAS FM DM HIT-6 Response rate Adverse reactions
Jinniu Li (2022) (49)	DECCME GTCM	China	Types of acupuncture: filiform needle Deqi achieved?: YES Acupuncturist background?: UN Needle retention time: 30 min Frequency of sessions and treatment courses: per cycle: acute (D-2 → pain resolution): qd Remission: q1-2d × 3 cycles Number of treatment sessions: UN	Western medicine (ibuprofen sustained-release capsule)	41; AGE: 19–40y (mean: 33 ± 7y); Disease duration: 6–240 m (mean: 55.6 ± 53.3 m)	42; AGE: 20–40y (mean: 32 ± 7y); Disease duration: 12-180 m (mean: 68.6 ± 44.6 m)	VAS Response rate
Rui Zhang (2022) (67)	ICHD-3 GTCM	China	Types of acupuncture: auricular point pressing bean Deqi achieved?: NO Acupuncturist background?: UN Needle retention time: 72 h Frequency of sessions and treatment courses: start D-7 to D-10: continuous × 15d (change q3d) per cycle × 3 cycles Number of treatment sessions: 15	Herbal medicine	30; AGE: 19–40y (mean: 33.1 ± 6.75y); Disease duration: -	30; AGE: 24–49y (mean: 32.9 ± 6.55y); Disease duration: -	Response rate Adverse reactions
Rui Li (2023) (68)	DECCME GTCM	China	Types of acupuncture: bloodletting at auricular dorsal vein + auricular point sticking Deqi achieved?: NO Acupuncturist background?: UN Needle retention time: 72 h Frequency of sessions and treatment courses: Bloodletting: D-7 start → q7d × 3 (alternate ears) Auricular pressing: D-7 start → q3d × 6 (alternate ears) Number of treatment sessions: 3 (Bloodletting), 6 (Auricular pressing)	Western medicine (flunarizine hydrochloride capsules)	48; AGE: 20–37y (mean: 27 ± 5y); Disease duration: 1–18y (mean: 6.9 ± 4.9y)	49; AGE: 21–38y (mean: 28 ± 5y); Disease duration: 1−20y (mean: 7.1 ± 5.8y)	MHI VAS 5-HT Response rate Adverse reactions
Guoliang Shao (2023) (69)	ICHD-3 DECCME GTCM	China	Types of acupuncture: auricular comprehensive therapy Deqi achieved?: NO Acupuncturist background?: UN Needle retention time: 0 min Frequency of sessions and treatment courses: start D-2 to D-5 pre-menses → q7d × 3 sessions Number of treatment sessions: 3	Western medicine (flunarizine hydrochloride capsules)	31; AGE: 23–39y (mean: 28 ± 3y); Disease duration: 1–16y (mean: 7.2 ± 3.9y)	31; AGE: 22–37y (mean: 27 ± 4y); Disease duration: 1–15y (mean: 7.9 ± 4y)	Response rate Adverse reactions
Wantao Wang (2023) (71)	ICHD-3 DECCME	China	Types of acupuncture: Jiejie acupuncture Deqi achieved?: YES Acupuncturist background?: UN Needle retention time: 30 min Frequency of sessions and treatment courses: onset → qwk × 4 sessions (1 course) × 2 courses Number of treatment sessions: 8	Western medicine (ibuprofen capsule)	50; AGE: (mean: 29.86 ± 7.27y); Disease duration: (mean: 5.06 ± 2.47y)	50; AGE: (mean: 31.16 ± 6.67y); Disease duration: (mean: 5.84 ± 2.08y)	VAS Response rate Adverse reactions
Lilan Chen (2024) (70)	DECCME	China	Types of acupuncture: auricular comprehensive therapy Deqi achieved?: NO Acupuncturist background?: UN Needle retention time: 72 h-192 h Frequency of sessions and treatment courses: onset → qwk × 4 sessions (1 course) × 2 courses Number of treatment sessions: 15	Western medicine (flunarizine hydrochloride capsules)	30; AGE: (mean: 30.9 ± 6.58y); Disease duration: -	30; AGE: (mean: 28.3 ± 6.86y); Disease duration: -	HIT-6
Ruolan Lv (2024) (50)	ICHD-3	China	Types of acupuncture: filiform needle Deqi achieved?: YES Acupuncturist background?: UN Needle retention time: 30 min Frequency of sessions and treatment courses: once Number of treatment sessions: 1	Western medicine (ibuprofen capsule)	30; AGE: (mean: 24.4 ± 2.74y); Disease duration: (mean: 5.23 ± 2.83y)	30; AGE: (mean: 24.73 ± 2.5y); Disease duration: (mean: 5.63 ± 2.4y)	VAS Adverse reactions
Yang Yang (2024) (62)	ICHD-3	China	Types of acupuncture: warm acupuncture Deqi achieved?: YES Acupuncturist background?: UN Needle retention time: 30 min Frequency of sessions and treatment courses: D-7 pre-menses → qd × 7d per cycle × 3 cycles Number of treatment sessions: 21	Western medicine (flunarizine hydrochloride capsules)	37; AGE: 20−45y (mean: 37.87 ± 5.64y); Disease duration: 1−7 m (mean: 4.89 ± 0.85 m)	37; AGE: 21−45y (mean: 37.97 ± 5.51y); Disease duration: 1−8 m (mean: 4.93 ± 0.71 m)	Response rate VAS FM DM
Huiling Ye (2024) (51)	ICHD-3 DECCME	China	Types of acupuncture: filiform needle Deqi achieved?: UN Acupuncturist background?: UN Needle retention time: 30 min Frequency of sessions and treatment courses: D-7 pre-menses → qd × 7d per cycle × 3 cycles Number of treatment sessions: 21	Western medicine (flunarizine hydrochloride capsules)	30; AGE: (mean: 34.07 ± 6.119y); Disease duration: (mean: 25 m)	30; AGE: (mean: 33.43 ± 6.426y); Disease duration: (mean: 24 m)	Response rate Adverse reactions
Tianyi Zhang (2024) (72)	ICHD-3 DTCDIMTCM GDTCDGTCM	China	Types of acupuncture: eye acupuncture Deqi achieved?: UN Acupuncturist background?: UN Needle retention time: 30 min Frequency of sessions and treatment courses: D-5 pre-menses → qd × 7d per cycle × 4 cycles Number of treatment sessions: 28	Western medicine (ibuprofen capsule)	30; AGE: (mean: 32.13 ± 4.76y); Disease duration: (mean: 11.27 ± 5.21y)	30; AGE: (mean: 31.77 ± 4.45y); Disease duration: (mean: 11.9 ± 4.2y)	VAS HIT-6 Adverse reactions
Qiuyue Zhang (2024) (73)	ICHD-3 DECCME GTCM	China	Types of acupuncture: electroacupuncture Deqi achieved?: YES Acupuncturist background?: UN Needle retention time: 25 min Frequency of sessions and treatment courses: D4 menses → q2d (3×/wk) × 4wk (12 sessions/cycle) × 2 cycles Number of treatment sessions: 24	Western medicine (ibuprofen capsule)	33; AGE: (mean: 29.12 ± 5.61y); Disease duration: (mean: 4.83 ± 2.14y)	33; AGE: (mean: 27.33 ± 4.75y); Disease duration: (mean: 4.73 ± 2.65y)	MHI Response rate Adverse reactions

CDCHDCNFP, Classification and Diagnostic Criteria for Headache Disorders, Cranial Neuralgias and Facial Pain; FM, Frequency of Migraine Attacks; VAS, Visual Analog Scale; DECCME, Diagnostic Efficacy Criteria for Chinese Medical Evidence; DM, Duration of Migraine; MHI, Menstrual Headache Index; ICHD-2, International Classification of Headache Disorders: 2nd edition; CBDEEH, Criteria for Both Diagnosis and Efficacy Evaluation of Headwind; DTRCMCTCM, Diagnosis and Treatment Routines for Common Medical Conditions in Traditional Chinese Medicine; OGITCWM, Obstetrics and Gynecology in Integrated Traditional Chinese and Western Medicine; HIT-6, Headache Impact Test-6; ICHD-3, International Classification of Headache Disorders, 3rd edition; 5-HT, 5-hydroxytryptamine; GTCM, Gynecology of Traditional Chinese Medicine; CGDTM, Chinese Guidelines for the Diagnosis and Treatment of Migraine; COG, Chinese Obstetrics and Gynecology; OG, Obstetrics and Gynecology; GPCRNCM, Guiding Principles for Clinical Research of New Chinese Medicines; DTCDIMTCM, Diagnosis and Treatment of Common Diseases in Internal Medicine of Traditional Chinese Medicine; GDTCDGTCM, Guidelines for Diagnosis and Treatment of Common Diseases of Gynecology in Traditional Chinese Medicine; UN, Unclear.

As presented in Table 1, different studies have used different diagnostic criteria for the definition and diagnosis of MM.

The reported duration of acupuncture intervention in the 39 RCTs varied, ranging from a single session (50) to a six-month course (56).

In terms of control group design, 26 of the studies (36, 37, 39, 44, 45, 49–55, 57–59, 61, 62, 64–66, 68–73) that employed Western medicine interventions as control conditions, all utilized orally administered medications. Six studies (38, 46, 56, 60, 63, 67) used Traditional Chinese Medicine as the control, and seven studies (35, 40–43, 47, 48) adopted sham acupuncture as the control. Western medicine included ibuprofen, aspirin, Celebrex, and flunarizine hydrochloride. The duration of treatment ranged from 1 (50) to 6 months (56) in the control group.

### Risk of bias in the included studies

**Figure 2:**
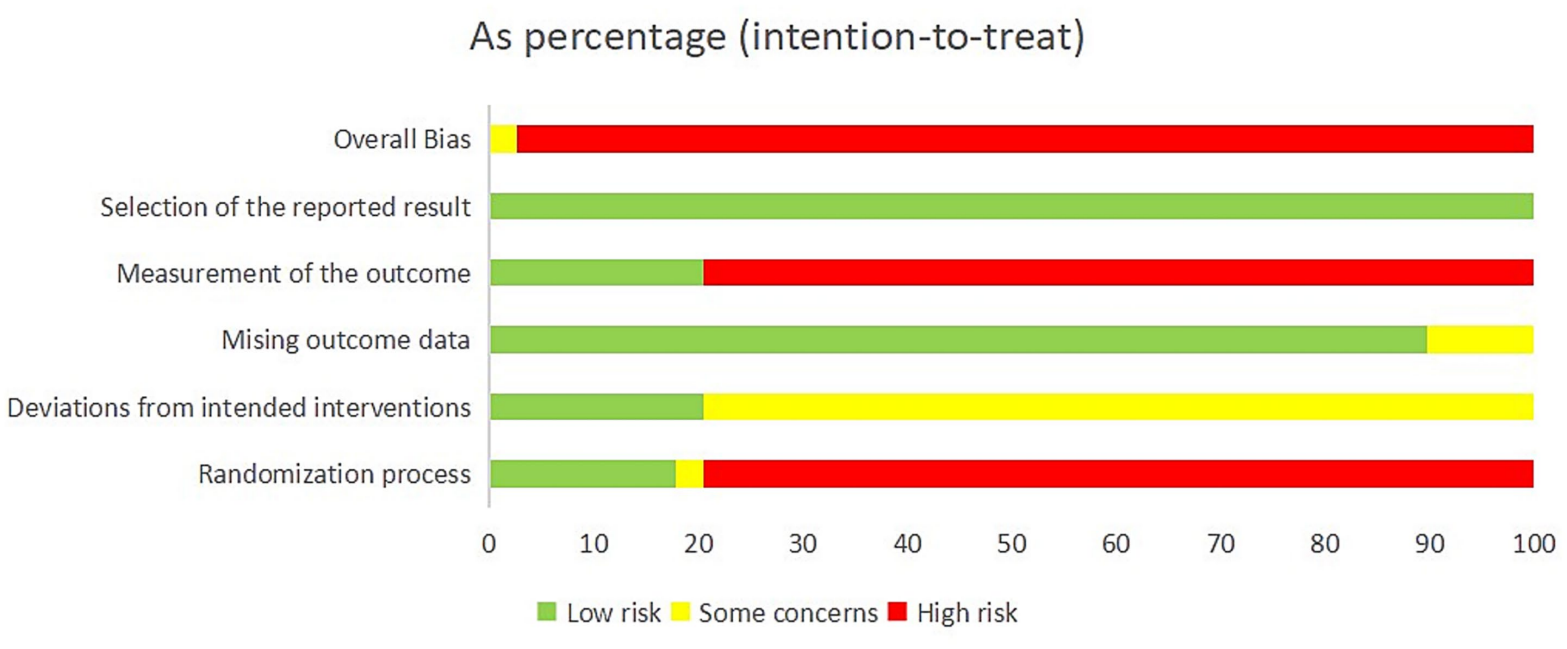
Results of quality assessment of included RCTs. Potential risk of bias for each included study. Summarized risk of included studies. RCT, randomized controlled trials.

Of the 39 included studies, the risk of bias was assessed according to the Cochrane RoB 2.0 tool, with the following results across each domain (Figure 2).

#### Bias arising from the randomization process

Seven studies that implemented appropriate random sequence generation and allocation concealment were rated as “low risk.” Thirty-one studies were judged to be at “high risk” due to the absence of allocation concealment. One additional study that used enrollment order as a quasi-random method despite having allocation concealment was rated as having “some concerns.”

#### Bias due to deviations from intended interventions

Eight studies that utilized sham acupuncture controls were rated “low risk.” The remaining 31 studies were rated as having “some concerns” due to the lack of blinding of participants and personnel, and the potential impact of knowledge of intervention assignment on adherence and co-interventions.

#### Bias due to missing outcome data

Five studies with dropouts that did not provide reasons or perform appropriate intention-to-treat analysis were rated as having “some concerns.” The remaining studies were considered “low risk” due to either no missing data or appropriate handling of missing outcomes.

#### Bias in measurement of the outcome

Eight studies that blinded outcome assessors were rated “low risk.” The other 31 studies were rated “high risk” due to lack of blinding of outcome assessment, which may have influenced the interpretation of subjective outcomes.

#### Bias in selection of the reported result

All studies were rated “low risk,” as no evidence of selective outcome reporting was identified.

### Overall risk of bias

Thirty-eight studies were judged to be at “high risk” of overall bias, primarily due to concerns across multiple domains including randomization, deviations from intended interventions, and measurement of outcomes. Rui Li (68) was rated as having “some concerns” overall.

### The efficacy and safety of acupuncture for treating MM

#### Pain intensity (VAS)

**Figure 3:**
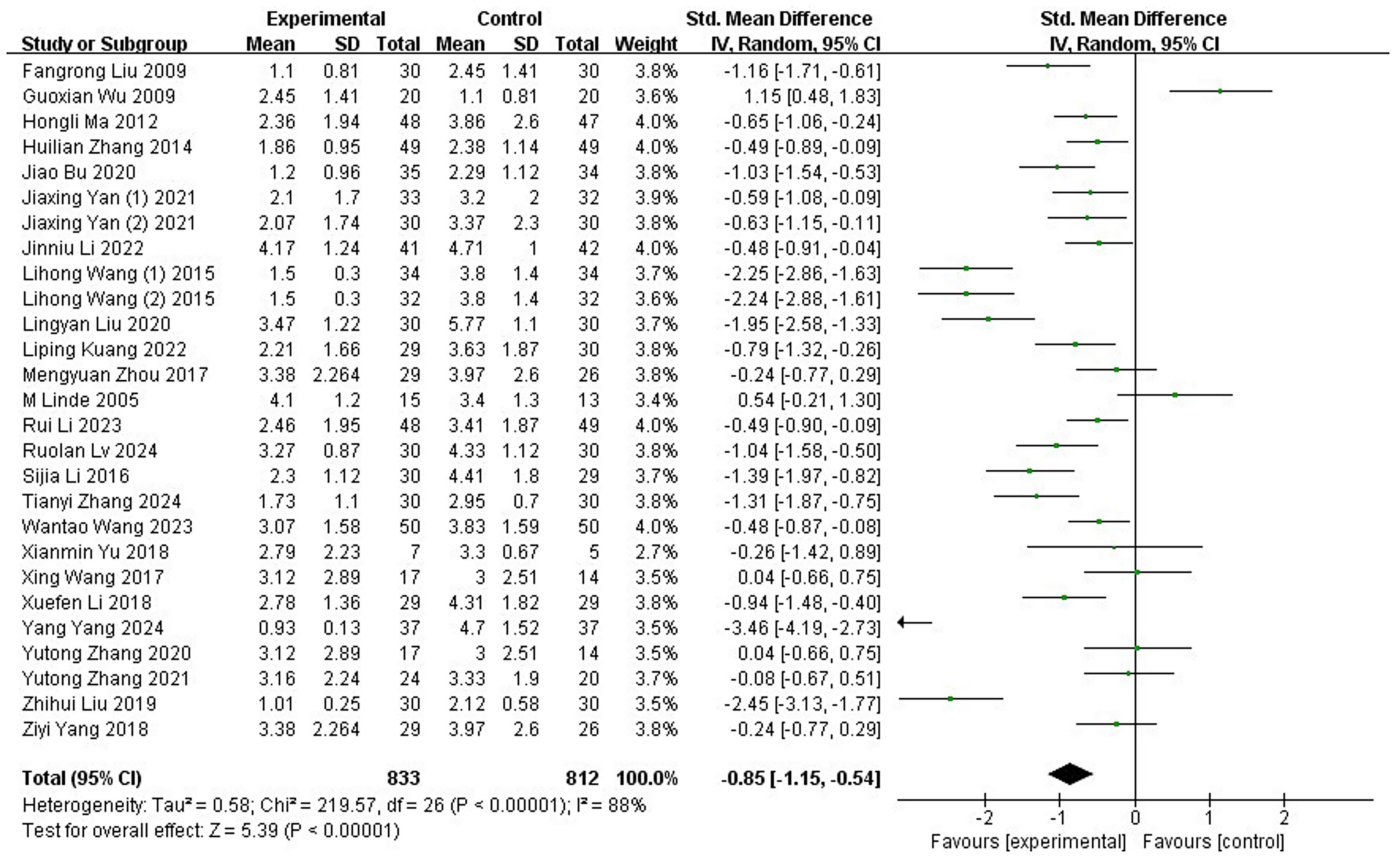
Meta-analysis of VAS data. VAS, Visual Analog Scale.

A total of 27 studies (35, 37, 38, 40–44, 46–50, 53, 56–62, 64–66, 68, 71, 72), involving 1,645 participants, employed the VAS to evaluate the severity of migraine. Substantial heterogeneity was detected across these trials, as indicated by the chi-squared test (*p* < 0.00001, I^2^ = 88%). Consequently, a REM was applied for the meta-analysis. The results showed that the acupuncture group reported significantly lower VAS scores compared to the control group (SMD: −0.85; 95% CI: [−1.15, −0.54]; Z: 5.39; *p* < 0.00001, Figure 3).

**Figure 4:**
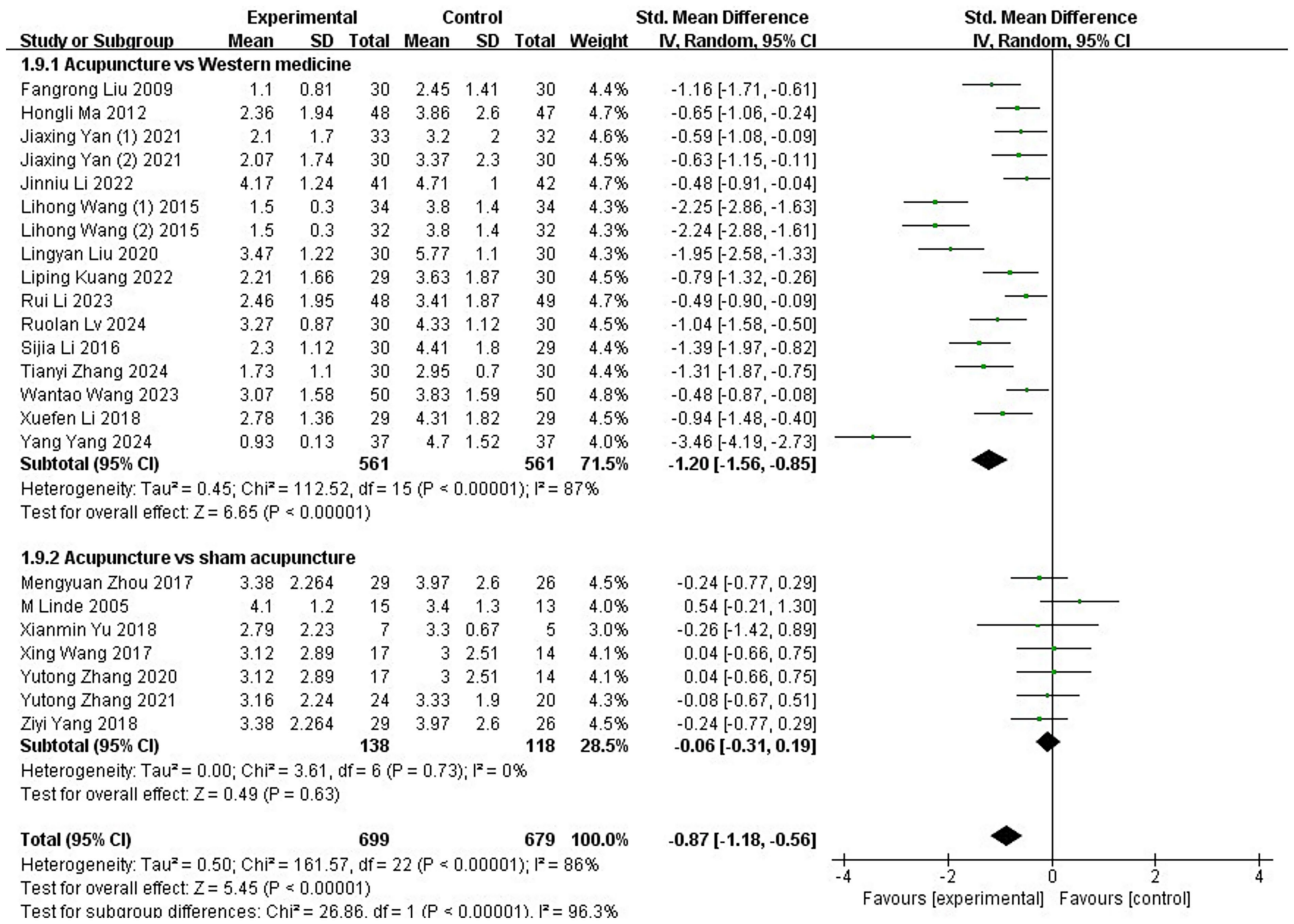
Subgroup analysis based on different interventions in the control groups.

Subgroup analyses were also performed for this outcome. When stratified by control type, interventions were categorized into Western medicine and sham acupuncture. Subgroup analysis revealed that acupuncture was significantly more effective than Western medicine (SMD: −1.2; 95% CI: [−1.56, −0.85]; Z = 6.65; *p* < 0.00001). However, no significant difference was found between acupuncture and sham acupuncture (SMD: −0.06; 95% CI: [−0.31, 0.19]; Z = 0.49; *p* = 0.63; Figure 4).

**Figure 5:**
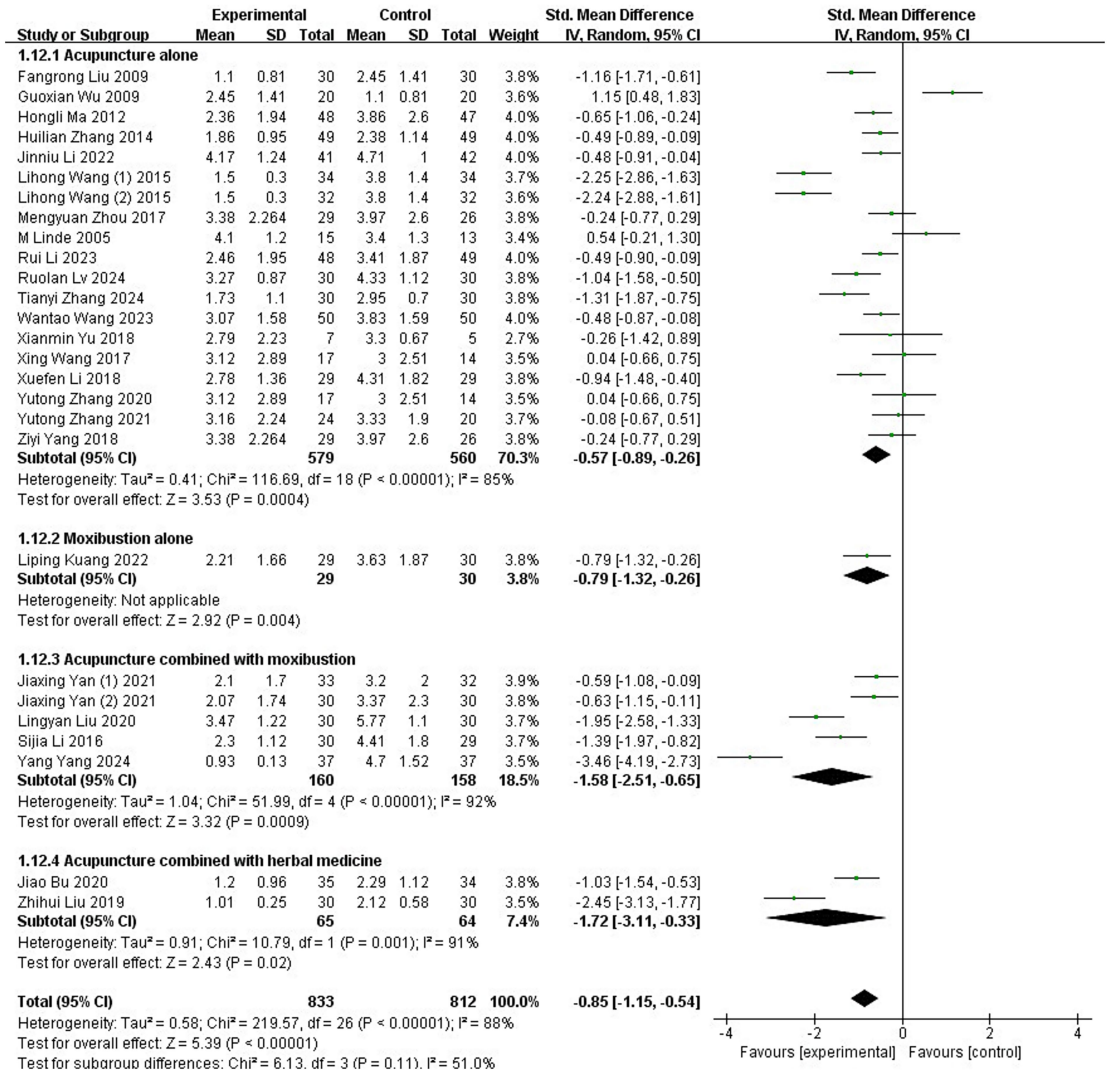
Subgroup analysis based on different interventions in the experimental groups.

A subgroup analysis was conducted to compare the VAS of different acupuncture-related modalities for MM management. Studies were stratified into four distinct subgroups: (1) acupuncture alone, (2) moxibustion alone, (3) acupuncture combined with moxibustion, and (4) acupuncture combined with herbal medicine. The analysis demonstrated that all four intervention modalities resulted in significantly greater reductions in VAS scores compared to control groups (1) SMD: −0.57; 95% CI: [−0.89, −0.26]; Z: 3.53; *p* = 0.0004; (2) SMD: −0.79; 95% CI: [−1.32, −0.26]; Z: 2.92; *p* = 0.004; (3) SMD: −1.58; 95% CI: [−2.51, −0.65]; Z: 3.32; *p* = 0.0009; (4) SMD: −1.72; 95% CI: [−3.11, −0.33]; Z: 2.43; *p* = 0.02, respectively, Figure 5, indicating superior effectiveness in alleviating migraine pain intensity across all approaches.

**Figure 6:**
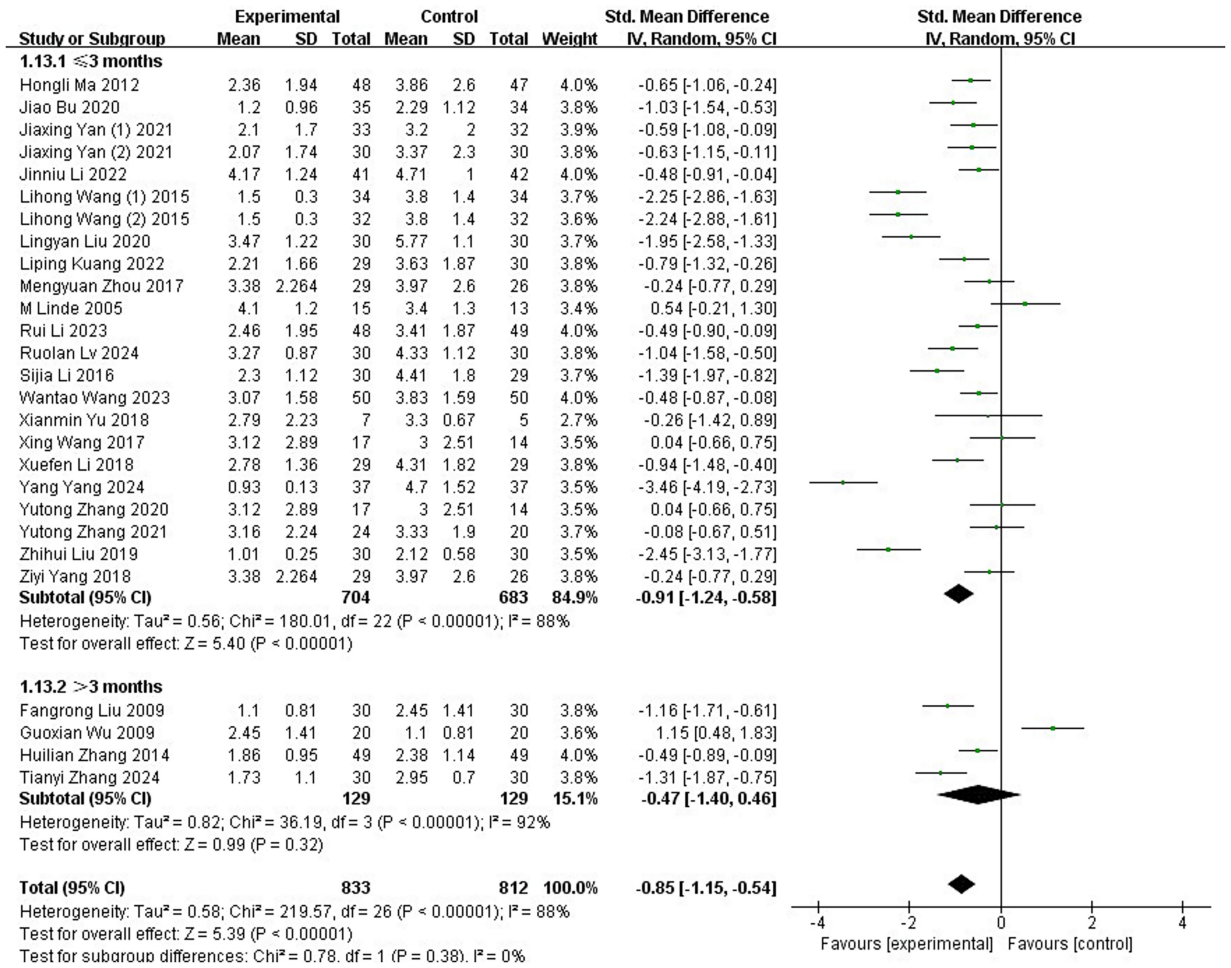
Subgroup analysis based on different treatment courses.

A subgroup analysis was conducted to assess whether the duration of acupuncture treatment influenced its VAS on MM. Studies were categorized into two subgroups based on treatment duration: ≤3-month interventions and >3-month interventions. The analysis revealed a statistically significant advantage for the ≤3-month intervention group (SMD: −0.91; 95% CI: [−1.24, −0.58]; Z: 5.40; *p*<0.00001), indicating substantial reduction in migraine pain intensity. In contrast, interventions > 3 months showed no statistically significant benefit over control conditions (SMD: −0.47; 95% CI: [−1.40, 0.46]; Z: 0.99; *p* = 0.32, Figure 6). This suggests that extending acupuncture treatment beyond 3 months may not provide additional therapeutic value for menstrual migraine management.

#### FM

**Figure 7:**
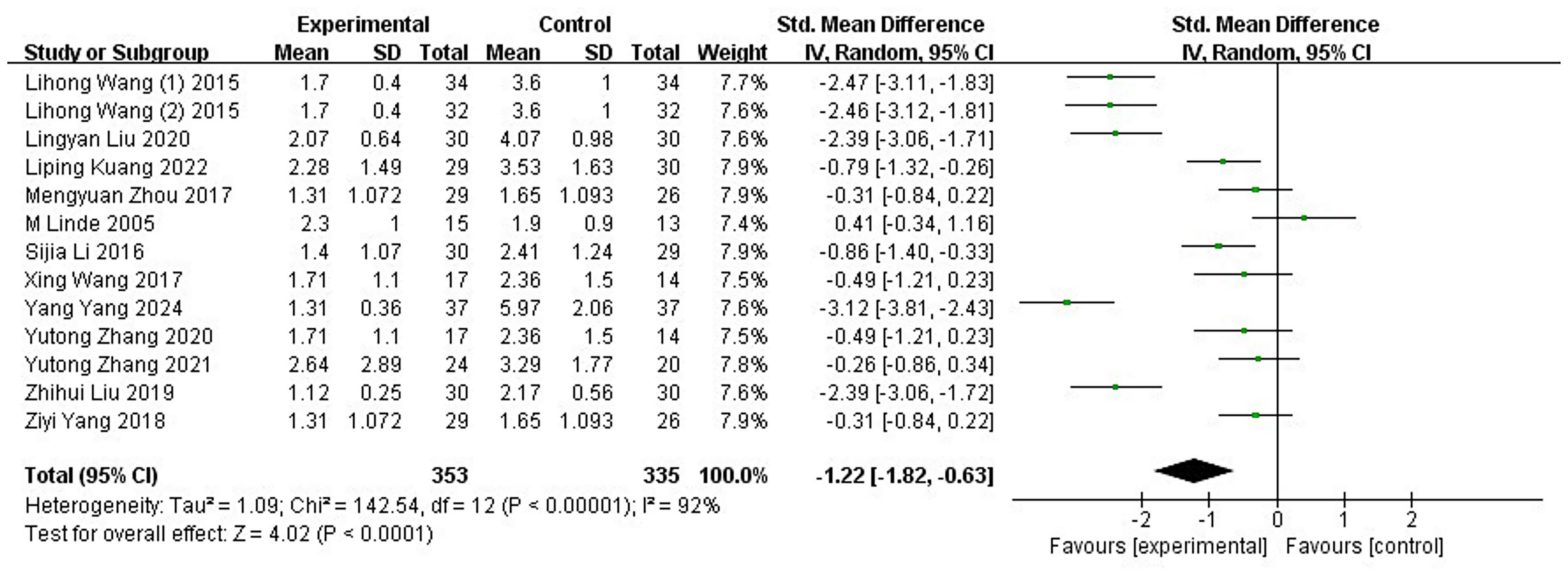
Meta-analysis of FM data. FM, the frequency of migraine attacks.

Thirteen studies (35, 40–42, 46–48, 57–59, 61, 62, 66), encompassing 688 participants, reported frequency measurements of MM using FM. The data exhibited substantial heterogeneity (*p* < 0.00001, I^2^ = 92%), prompting the application of a REM for analysis. Meta-analysis of the combined results demonstrated that participants receiving acupuncture showed significantly reduced FM values compared to those in the control group (SMD: −1.22; 95% CI: [−1.82, −0.63]; Z: 4.02; *p* < 0.0001, Figure 7).

#### DM

**Figure 8:**
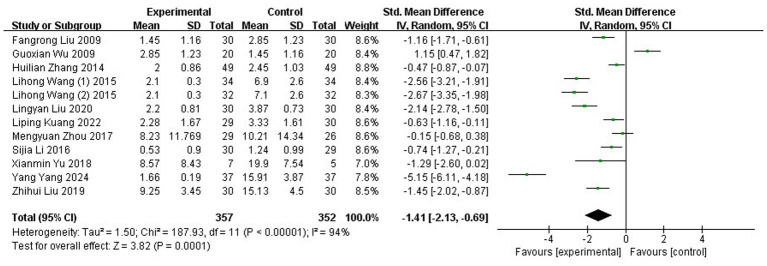
Meta-analysis of DM data. DM, duration of migraine.

Twelve RCTs (37, 38, 41, 43, 46, 56–59, 61, 62, 66), involving a total of 709 patients diagnosed with MM, reported data on DM. Considerable heterogeneity was detected among the studies (*p* < 0.00001, I^2^ = 94%), warranting the use of a REM for meta-analysis. The pooled results revealed a statistically significant reduction in DM in the acupuncture group compared to controls (SMD: −1.41; 95% CI: [−2.13, −0.69]; Z: 3.82; *p* = 0.0001), suggesting that acupuncture may effectively improve DM levels (Figure 8).

#### Response rate

**Figure 9:**
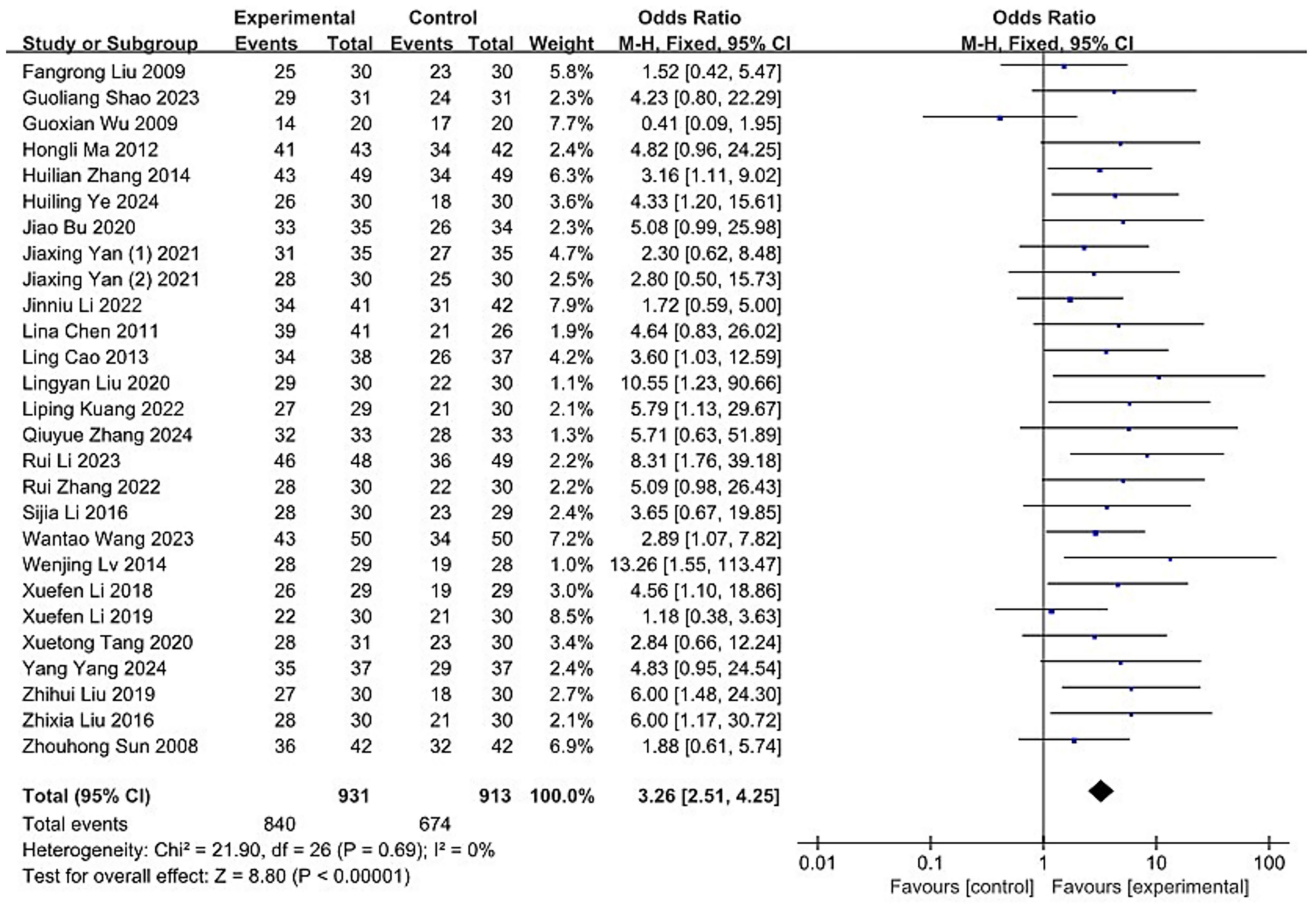
Forest plot and meta-analysis of the response rate of acupuncture in the treatment of MM. MM, menstrual migraine.

The response rate of acupuncture for MM was evaluated in 27 RCTs (36–39, 44–46, 49, 51–56, 59–69, 71, 73), encompassing a total of 1,844 participants. The acupuncture group demonstrated a total effective rate of 90.23% (840/931), whereas the control group achieved 73.82% (674/913). Meta-analytic results indicated a statistically significant advantage for acupuncture over control interventions (OR: 3.26; 95% CI: [2.51, 4.25]; Z: 8.8; *p* < 0.00001, Figure 9). There was no indication of substantial heterogeneity among the included trials (χ2 = 21.9; *p* = 0.69; I^2^ = 0%).

#### HIT-6

**Figure 10:**
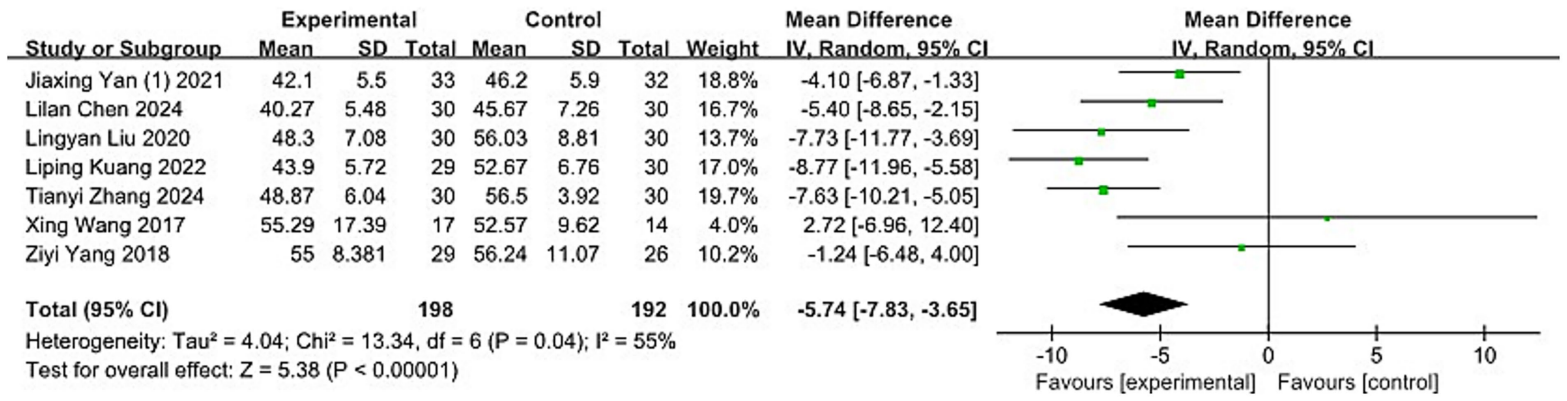
Meta-analysis of HIT-6 data. HIT-6, Headache Impact Test-6.

Seven studies (40, 42, 61, 64, 66, 70, 72), encompassing 390 participants, employed the HIT-6 scale to evaluate the impact of headaches. The Chi-squared test indicated moderate heterogeneity between the studies (*p* = 0.04, I^2^ = 55%), leading to the application of a REM for analysis. The meta-analysis showed that acupuncture significantly reduced headache impact compared to the control group (MD: −5.74; 95% CI: [−7.83, −3.65]; Z: 5.38; *p* < 0.00001, Figure 10).

#### MHI

**Figure 11:**
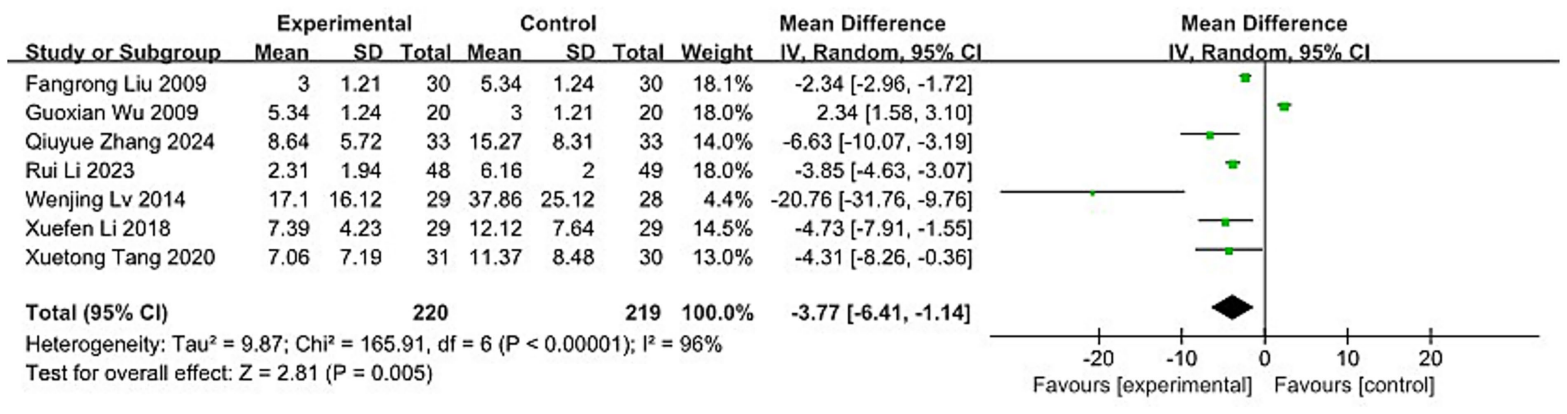
Meta-analysis of MHI data. MHI, the menstrual headache index.

Seven trials (37, 38, 44, 55, 63, 68, 73), involving a total of 439 participants, assessed the effect of acupuncture on MM with associated pain index. The Chi-squared test revealed high heterogeneity between studies (*p* < 0.00001, I^2^ = 96%), prompting the use of a REM for analysis. Meta-analysis indicated that acupuncture was significantly more effective than the control intervention (MD: −3.77; 95% CI: [−6.41, −1.14]; Z: 2.81; *p* = 0.005, Figure 11).

#### 5-HT

**Figure 12:**
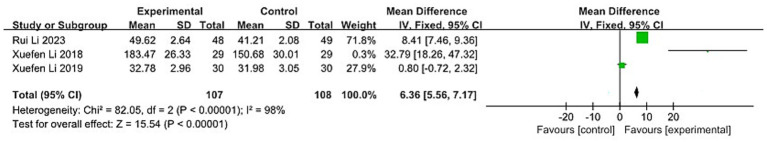
Meta-analysis of 5-HT data. 5-HT, serum concentrations of 5-hydroxytryptamine.

Three trials (44, 45, 68), comprising 215 participants, evaluated serum 5-HT levels to assess treatment effects. Substantial heterogeneity was identified between the studies (*p* < 0.00001, I^2^ = 98%), necessitating the use of a REM. Meta-analysis results indicated that acupuncture significantly reduced serum 5-HT levels compared to the control group (MD: −6.36; 95% CI: [5.56, 7.17]; Z: 15.54; *p* < 0.00001, Figure 12). However, considering the modest magnitude of the effect, its clinical significance remains uncertain.

#### Adverse reactions

**Figure 13:**
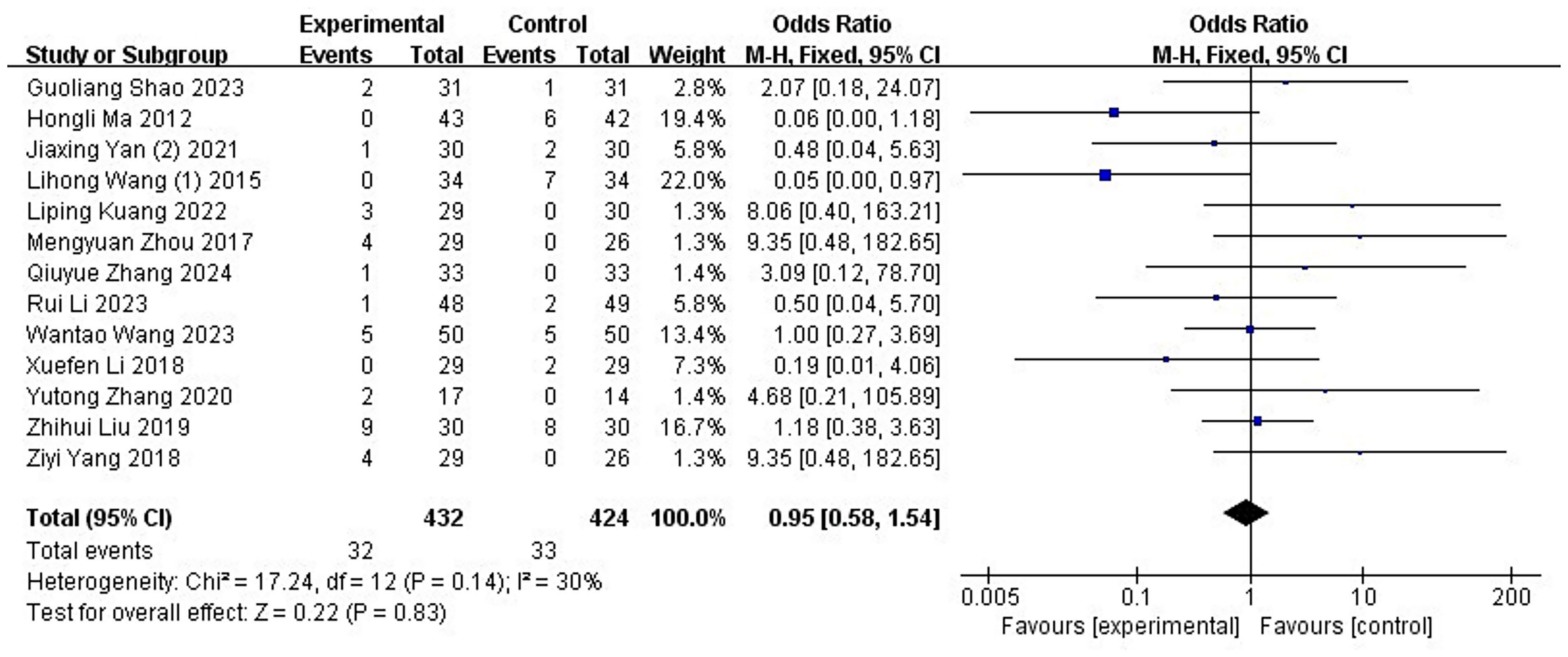
Meta-analysis of adverse reactions.

Adverse reactions of acupuncture for MM was evaluated in 13 RCTs (41, 42, 44, 46, 48, 53, 57, 65, 66, 68, 69, 71, 73), encompassing a total of 856 participants. The acupuncture group demonstrated a total adverse rate of 7.4% (32/432), whereas the control group achieved 7.8% (33/424). Meta-analytic results showed that the incidence of adverse events did not differ significantly between the acupuncture and control groups (OR: 0.95; 95% CI: [0.58, 1.54]; Z: 0.22; *p* = 0.83, Figure 13). There was no indication of substantial heterogeneity among the included trials (χ2 = 17.24; *p* = 0.14; I^2^ = 30%). Of the 39 RCTs, 21 studies reported adverse event data. Eight studies (50, 51, 55, 60, 61, 63, 67, 72) indicated no adverse reactions. Several trials involving Western medicine reported mild gastrointestinal side effects, including nausea, vomiting, drowsiness, and stomach discomfort. Acupuncture-related events included needle fainting, localized hematoma, bruising, and dizziness. Combined therapies (e.g., filiform needle with Chinese medicine or moxibustion) occasionally led to minor issues like diarrhea, skin swelling, hair singeing, or fainting, all of which resolved with simple treatment or patient reassurance. The remaining 18 studies did not mention adverse events.

### Sensitivity analyses

Sensitivity analyses were conducted by sequentially removing each individual study and recalculating the pooled standardized mean difference (SMD). The results indicated that the overall estimates were stable for the primary outcomes and not unduly influenced by any single study. For the VAS outcome, the pooled SMDs ranged from −0.75 (95% CI: −1.03, −0.48) to −0.92 (95% CI: −1.21, −0.63), all remaining statistically significant (*p* < 0.05). Similarly, the conclusions for FM and DM were unaltered, with all recalculated SMDs continuing to favor the acupuncture group. Therefore, the results confirmed that our findings were robust.

### Publication Bias

**Figure 14:**
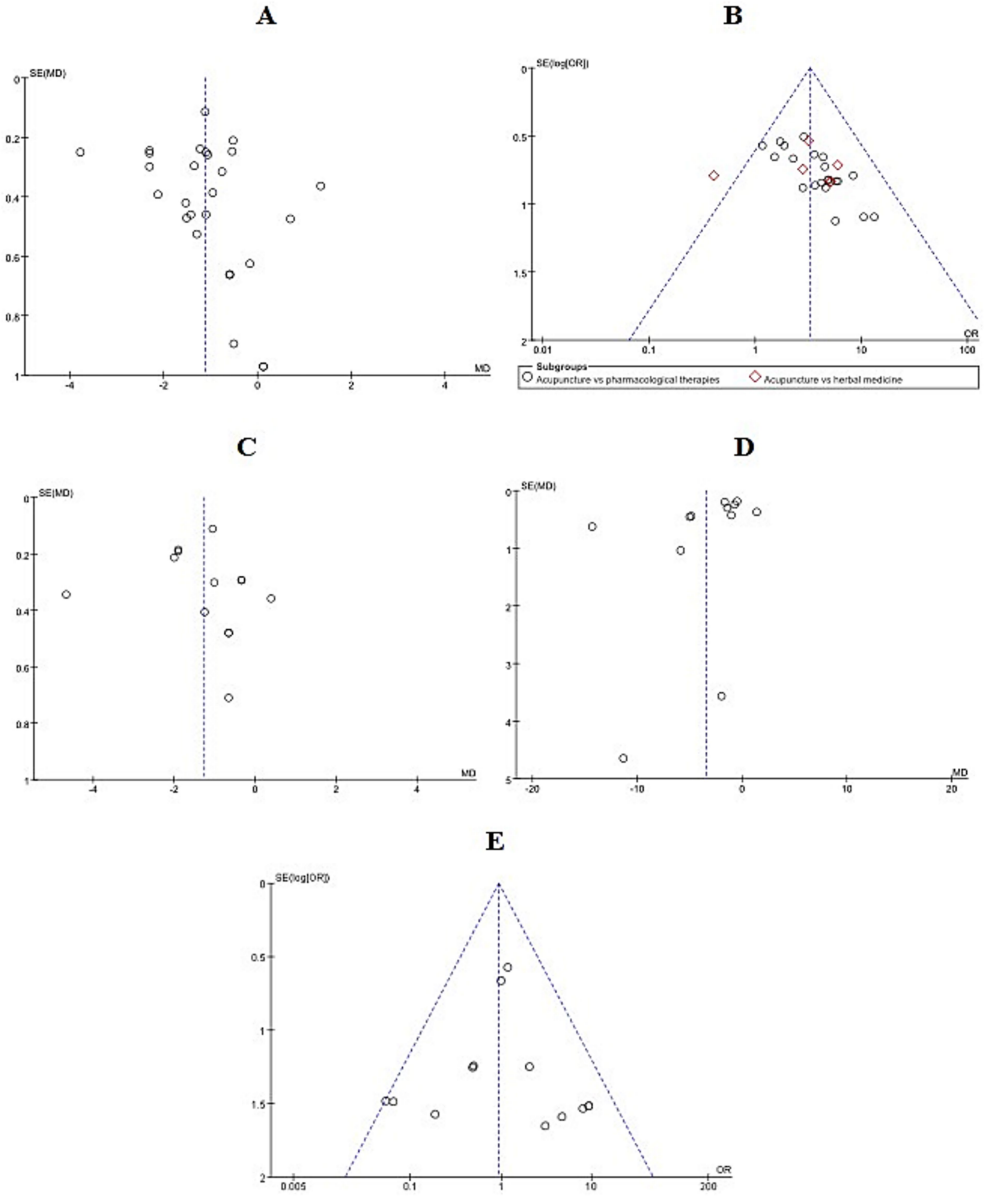
Funnel plot of publication bias. **(A)** VAS. **(B)** Response rate. **(C)** FM. **(D)** DM. **(E)** Adverse reactions. VAS, Visual Analog Scale; FM, the frequency of migraine attacks; DM, duration of migraine.

Funnel plots were used to assess publication bias when the number of studies included in an outcome measure was 10 or more. The plots for VAS, response rate and adverse reactions showed an approximately symmetrical distribution within the confidence boundaries, suggesting no evident publication bias (*p* > 0.05). In the response rate plot, most data points clustered toward the top indicated that the included studies had relatively large sample sizes. However, one point appeared outside the funnel on the left side, indicating a potential minor publication bias. In contrast, the funnel plots for FM and DM exhibited notable asymmetry, suggesting a higher likelihood of publication bias. This may be attributable to the small sample sizes and generally low methodological quality of the included trials, which increases the probability of selective publication of positive results (Figure 14). This visual assessment was supported by Egger’s test, which indicated significant asymmetry for FM and DM (*P* <0.05), but not for VAS, response rate, or adverse reactions (*P* < 0.05).

### GRADE assessment

Table 2 presents a summary of the evidence quality assessment for outcome measures included in 10 or more studies, evaluating the effects of acupuncture. The certainty of evidence varied across outcomes: low for VAS scores, moderate for response rate and adverse reactions, and very low for both FM and DM levels. Downgrades in evidence quality were attributed to a high risk of bias within the included studies, substantial heterogeneity in effect estimates, variability in interventions and study designs, and the presence of publication bias. These factors may significantly compromise the reliability and validity of the pooled findings.

**Table 2:** GRADE quality of evidence assessment of outcome indicators for the included studies.

Outcome/no. of studies	Design	Quality assessment					No. of patients		Relative (95% CI) absolute	Quality	Importance
		Risk of bias	Inconsistency	Indirectness	Imprecision	Publication bias	Acupuncture	Control			
VAS (*n* = 27)	Randomized trials	Serious^a^	Serious^b^	No serious indirectness	Serious^c^	Suspected^d^	833	812	MD = −1.11 (−1.5,−0.72)	⊕ ⊕ ○○Low	Crucial
FM (*n* = 13)	Randomized trials	Serious^a^	Serious^b^	No serious indirectness	Serious^c^	Strongly suspected^e^	353	335	MD = −1.26 (−1.81,−0.71)	⊕○○○Very low	Important
DM (*n* = 12)	Randomized trials	Serious^a^	Serious^b^	No serious indirectness	Serious^c^	Strongly suspected^e^	357	352	MD = −3.45 (−5.09,−1.81)	⊕○○○Very low	Important
Response rate (*n* = 27)	Randomized trials	Serious^a^	Not serious	No serious indirectness	Serious^c^	Suspected^d^	840/931 (90.23%)	674/913 (73.82%)	OR = 3.26 (2.51,4.25)	⊕ ⊕ ⊕○Moderate	Crucial
Adverse reactions (*n* = 13)	Randomized trials	Serious^a^	Not serious	No serious indirectness	Serious^c^	Suspected^d^	32/432 (7.4%)	33/424 (7.8%)	OR = 0.95 (0.58,1.54)	⊕ ⊕ ⊕○Moderate	Crucial

GRADE, Grading of Recommendations Assessment, Development, and Evaluation; VAS, Visual Analog Scale; FM, Frequency of Migraine Attacks; DM, Duration of Migraine; CI, Confidence Interval; MD, Mean Differences. ^a^Most RCTs at high risk of bias due to methodological limitations. ^b^Evidence of statistically significant interstudy heterogeneity. ^c^Interventions differed. ^d^The funnel plot is slightly asymmetrical. ^e^The funnel plot is clearly asymmetrical.

## Discussion

### Summary of results

This meta-analysis included 39 RCTs and assessed 7 outcome indicators. The findings indicate that acupuncture offers greater therapeutic benefits for MM compared to control interventions. Specifically, acupuncture led to notable reductions in migraine-related parameters, including intensity (VAS), frequency (FM), duration (DM), and overall migraine burden (MHI), suggesting a substantial mitigating effect on headache severity, frequency, and duration. Additionally, it significantly improved clinical response rates in patients with MM. Regarding headache impact, acupuncture significantly lowered HIT-6 scores, reflecting an improvement in patient quality of life. Furthermore, acupuncture was associated with elevated serum 5-HT levels, suggesting a potential role in modulating neurohormonal function.

The therapeutic benefits of acupuncture observed in this meta-analysis may be attributed to its neuromodulatory effects on the central nervous system. Acupuncture is thought to influence pain processing by modulating brain activity in key regions involved in migraine pathogenesis, such as the periaqueductal gray, thalamus, and somatosensory cortex (23, 24, 40, 42). This aligns with a growing body of evidence supporting non-pharmacological approaches for migraine management that target aberrant neural circuitry. A recent rigorous GRADE evaluation by Tana et al. (74) confirmed the efficacy and provided strong recommendations for certain invasive and non-invasive neuromodulation devices in chronic migraine. While their review focused on electronic devices and did not evaluate acupuncture, our findings position traditional acupuncture as a clinically effective, non-invasive modality that appears to share a common therapeutic goal with these advanced techniques: the direct modulation of the nervous system to restore normal function. This places acupuncture within a modern, evidence-based therapeutic paradigm beyond its traditional roots.

Overall, the evidence supports the superior response of acupuncture over control treatments in managing MM. Of note, the lack of significant difference between real and sham acupuncture in subgroup analysis in terms of VAS may stem from the partial therapeutic effect of the sham methods. Some sham protocols used needling near actual acupoints, yet modern research suggests acupoints are dynamic and may expand under pathological conditions. As a result, these “sham” points may still fall within sensitized regions, exerting clinical effects and blurring the distinction between real and sham acupuncture in outcomes.

### Safety analysis

In the acupuncture group, 28 patients experienced adverse events. These included 13 cases of bruising, which typically resolved without intervention within a few days. Additionally, two incidents of ash scattering and one instance of hair being singed by moxa fire were reported, underscoring the importance of maintaining proper technique and patient stability during treatment. Eight patients experienced fainting, potentially attributable to factors such as low pain tolerance, fasting, or undergoing acupuncture for the first time. Other reported effects included three cases of diarrhea and one case of nausea with vomiting, possibly linked to fasting, emotional stress, or the patient’s physical condition at the time of treatment. In the control group, 29 adverse events were documented, including two cases presented with weakness and drowsiness, one with fatigue, five with nausea and gastrointestinal reactions, three with vomiting, four with drowsiness, one with mild nausea, two with stomach burning or ache, two with localized hematoma, one with diarrhea, one with fainting, one with loss of appetite, one with nausea and vomiting, and five with stomach pains. These reactions may be associated with patients’ baseline health status or adverse effects of Western medicine. Overall, the meta-analysis suggests that acupuncture presents a favorable safety profile in the management of MM.

### Quality of the evidence

These findings should be interpreted with caution, as many of the included RCTs exhibited a high risk of bias. Notably, concerns arise from inadequate random sequence generation, as well as deficiencies in allocation concealment and blinding procedures. Proper randomization methods—such as using random number tables—are essential for minimizing selection bias. Likewise, allocation concealment and participant or assessor blinding are critical to ensure methodological rigor. Evidence suggests that trials with unclear or inadequate allocation concealment may overestimate treatment effects by an average of 18% (95% CI: 5 to 29%) (30). Additionally, a meta-epidemiological analysis found that unblinded trials produced effect sizes 0.56 standard deviations larger than those with proper blinding (75). In this review, the risk of bias in these domains remains substantial: only 8 studies adequately reported allocation concealment, and only 8 trials implemented blinding procedures.

### Limitations

First, the high heterogeneity observed in primary outcomes was not fully explored. While we attempted to conduct subgroup analyses to explore the sources of heterogeneity in terms of different modalities of acupuncture and different durations of acupuncture treatment, other potential sources of heterogeneity, such as different acupuncture protocols, disease durations and baseline patient characteristics, were not assessed due to insufficient reporting in the included studies. Second, the majority of trials exhibited a high risk of bias, particularly regarding the blinding of participants and practitioners, raising concerns about performance and detection bias. Third, response rate—a commonly reported outcome in Chinese RCTs—was used as a primary endpoint in this analysis. However, this measure is not universally accepted, limiting the comparability of our findings with other trials and SRs. Moreover, inconsistent definitions and calculation standards for “response rate” across studies further undermine the validity and reliability of this outcome (76). Therefore, results related to response rates should be interpreted with caution. Fourth, the vast majority of the included trials were conducted in China and published in Chinese. This raises the likelihood of publication bias, particularly given the disproportionately high rate of positive findings reported in acupuncture trials from China. Fifth, the number of trials reporting objective outcome measures was limited, constraining our ability to include more rigorous endpoints in the analysis.

### Implications for future studies

Drawing from the results of this meta-analysis, future studies are encouraged to prioritize the following areas. First, there is a need to establish standardized diagnostic definitions and unified outcome assessment tools. Second, stratification of MM by baseline factors—such as symptom severity and patient age—should be implemented to allow for more precise subgroup analyses. Third, acupuncture treatment protocols—including needling techniques, acupoint combinations, and instrument selection—should be categorized and systematized. This would enable meaningful comparisons and contribute to the development of a standardized acupuncture protocol rooted in Traditional Chinese medicine pattern differentiation. Lastly, recent studies (40, 42, 47, 48) have highlighted that functional abnormalities in the brain may underlie headaches and mood disturbances in MM patients, as revealed by functional magnetic resonance imaging (fMRI), when compared with healthy controls. Therefore, fMRI may serve as a promising tool for objectively evaluating therapeutic outcomes and refining diagnostic criteria for MM, ultimately enhancing the reliability and precision of research findings.

## Conclusion

This updated meta-analysis assessed the safety and effectiveness of acupuncture for managing MM and found that acupuncture significantly reduced migraine severity and improved quality of life. However, substantial heterogeneity across several pooled analyses highlights the urgent need for high-quality, large-scale RCTs to confirm these findings.

## Data Availability

The original contributions presented in the study are included in the article/Supplementary material, further inquiries can be directed to the corresponding authors.
