# Supplementary material for: Determining the efficacy and safety of acupuncture for the treatment of menstrual migraine: an updated systematic review and meta-analysis
Source: Front Neurol. 2025 Sep 17;16:1673321. doi: 10.3389/fneur.2025.1673321 (PMC12484189; doi:10.3389/fneur.2025.1673321)
Supplement: Supplementary file 1 [file Table_1.DOCX]

**Table 1** The search strategies for PubMed

| **No.** | **Search Items** |
| --- | --- |
| #1 | Randomized controlled trial [pt] |
| #2 | Controlled clinical trial [pt] |
| #3 | Randomized OR Randomised [Title/Abstract] |
| #4 | Clinical trials [MeSH] |
| #5 | Randomly [Title/Abstract] |
| #6 | Trial [Title/Abstract] |
| #7 | #1 OR #2 OR #3 OR #4 OR #5 OR #6 |
| #8 | Migraine disorders [MeSH] |
| #9 | (Migraine OR Menstrual migraine OR Menstrual OR Menstrually OR Menses OR Menstruation-related migraine OR Menstruation) [Title/Abstract] |
| #10 | #8 OR #9 |
| #11 | Acupuncture Therapy [MeSH] |
| #12 | (acupuncture OR manual acupuncture OR electro-acupuncture OR electroacupuncture OR acupoint injection OR plum blossom needling OR bloodletting OR pricking blood OR triangular needle OR intradermal needle OR acupoint catgut embedding OR fire acupuncture OR warming-needle moxibustion OR warm needle OR ear acupuncture OR auricular acupuncture OR scalp acupuncture) [Title/Abstract] |
| #13 | #11 OR #12 |
| #14 | #7 AND #10 AND #13 |
